# The Drp1-CoQ10-Coa6-ETC axis represents a therapeutic potential for working memory impairment caused by neuronal mitochondrial dysfunction

**DOI:** 10.1186/s40035-026-00552-6

**Published:** 2026-04-27

**Authors:** Jingjing Tie, Shujiao Li, Xin Huang, Keke Ren, Ziwei Ni, Xiaodong Li, Changlei Zhu, Hui Liu, Feifei Wu, Yanling Yang, Yayun Wang

**Affiliations:** 1 Specific Lab for Mitochondrial Plasticity Underlying Nervous System Diseases, The Fourth Military Medical University, Xi’an, 710032 China; 2 Department of Hepatobiliary Surgery, Xi-Jing Hospital, The Fourth Military Medical University, Xi’an, 710032 China; 3 Department of Hepatobiliary and Pancreatic Surgery, Affiliated Hospital of Yan’an University, Yan’an University, Yan’an, 716000 China; 4 Department of Laboratory Medicine, Xi’an Da-Xing Hospital, Xi’an, 710032 China; 5 Department of Human Anatomy, Histology and Embryology, Medical School of Yan’an University, Yan’an University, Yan’an, 716000 China

**Keywords:** Purkinje cells, Working memory, Coenzyme Q10, Dynamin-related protein 1, Cytochrome *c* oxidase assembly factor 6

## Abstract

**Background:**

Coenzyme Q10 (CoQ10) is a key mitochondrial electron carrier and a widely used dietary supplement with potential neurological benefits. However, the mechanisms underlying its effect in ameliorating memory deficits caused by cerebellar injury are not fully understood. In this study, we investigated the effects of long-term CoQ10 supplementation on working memory and the underlying mechanisms.

**Methods:**

Network pharmacology analysis was used to identify genetic targets of CoQ10 in cerebellar injury-related cognitive impairment. Purkinje cell (PC)-specific Drp1-deficient mice (PC-Drp1^−/−^) were generated to model mitochondrial dysfunction. Behavioral performance was evaluated using the eight-arm radial maze. Mitochondrial structure and respiratory chain complex levels were evaluated by morphological and biochemical assays. Molecular targets of CoQ10 were identified using integrated drug–target engagement approaches, and their functional relevance was tested by viral vector-mediated overexpression.

**Results:**

The PC-Drp1^−/−^ mice displayed progressive working memory impairment and decreased PC density, accompanied by disrupted mitochondrial morphology and reduced activities of electron transport chain complexes III–V. Long-term CoQ10 treatment significantly reduced working memory errors and preserved PC numbers in PC-Drp1^−/−^ mice. Target engagement analyses identified cytochrome c oxidase assembly factor 6 (Coa6) as a direct binding target of CoQ10. Viral vector-mediated overexpression of Coa6 in PCs partially recapitulated the CoQ10-associated improvements in respiratory chain complex levels and working memory, whereas Coa6 knockdown attenuated these benefits.

**Conclusions:**

CoQ10 directly interacts with Coa6 to enhance mitochondrial respiratory chain function and preserve PC integrity in the context of Drp1 deficiency. Our findings suggest a promising mechanistic pathway for CoQ10-based intervention in memory deficits associated with mitochondrial dysfunction.

**Supplementary Information:**

The online version contains supplementary material available at 10.1186/s40035-026-00552-6.

## Introduction

Neurological diseases constitute a growing proportion of the global disease burden [1]. Working memory refers to the temporary retention and manipulation of relevant information, which guides future actions [2]. Impairment of working memory is a common feature of multiple neurodegenerative disorders, including Alzheimer’s disease (AD), Parkinson’s disease (PD), and Huntington’s disease (HD). Despite its clinical relevance, effective therapeutic strategies targeting working memory dysfunction remain limited [3, 4]. Previous studies on working memory mainly focused on the cerebral cortex and hippocampus, while ignoring the role of the cerebellum [5]. However, accumulating evidence has redefined the cerebellum as a critical node in cognitive networks [6]. The cerebellum can affect the accurate maintenance of information in working memory [7]. During the accumulation process of sensory information, inhibition of the cerebellar nucleus caused by activation of Purkinje cells (PCs) reduces the accuracy of decisions made based on working memory. Wagner MJ et al. reported that cerebellar PCs, the sole output of the cerebellar cortex, are anatomically and functionally integrated into the prefrontal-cerebellar-thalamic circuits that have been implicated in higher-order cognitive processing, including working memory [8]. Nevertheless, the cellular and molecular mechanisms by which cerebellar PCs influence working memory remain incompletely understood.

Mitochondrial integrity is critical for neuronal function due to the high energetic demands for synaptic transmission and plasticity [9]. Disruptions of mitochondrial dynamics, bioenergetics, and redox homeostasis have been increasingly linked to the cognitive decline in neurodegenerative conditions [10]. Dynamin-related protein 1 (Drp1), a key regulator of mitochondrial fission, plays an essential role in maintaining mitochondrial structure and function in neurons [11]. Dysregulation of Drp1-mediated mitochondrial dynamics has been associated with synaptic dysfunction and neuronal vulnerability, highlighting mitochondrial homeostasis as a potential determinant of cognitive impairment [12]. Given the central role of mitochondria in neuronal energy metabolism and redox balance, mitochondria-targeted interventions have been proposed as potential strategies for ameliorating cognitive dysfunction [13]. Moreover, coenzyme Q10 (CoQ10) treatment effectively rescued mitochondrial dysfunction caused by PC-specific knockout of COQ8A [14]. In this context, CoQ10, an essential component of the mitochondrial electron transport chain (ETC), represents a biologically plausible candidate for modulating mitochondrial dysfunction associated with cerebellar-related cognitive impairment [15].

CoQ10 is an endogenous lipid-soluble antioxidant that participates in the mitochondrial oxidative phosphorylation (OXPHOS) process [16, 17]. Due to its roles in electron transfer and redox regulation, CoQ10 has attracted considerable interest as a neuroprotective agent and is widely used as a dietary supplement [16, 18]. Previous studies have reported beneficial effects of CoQ10 supplementation in models of neurodegenerative diseases and mitochondrial encephalopathies, including conditions involving cerebellar pathology [14, 19]. However, whether CoQ10 directly engages mitochondrial proteins involved in ETC assembly, rather than acting solely as a redox carrier, remains poorly explored [20, 21]. Therefore, whether CoQ10 can ameliorate working memory impairment associated with cerebellar injury, and through which mitochondrial mechanisms it exerts its effects, remain open questions. Addressing this gap may provide new insights into the mitochondrial regulation of cerebellar-dependent cognitive function and the development of targeted therapeutic strategies.

In this study, we first established the PC-specific Drp1-deficient mouse model and observed mitochondrial abnormalities, reduced PC number and altered dendritic morphology, along with impaired cerebellar-dependent working memory. We then evaluated the effects of CoQ10 treatment in this model and found that CoQ10 partially alleviated these mitochondrial and cellular alterations as well as the associated working memory deficits. Based on these observations, we subsequently performed protein-based screening and identified cytochrome c oxidase assembly factor 6 (Coa6) as a mitochondrial protein associated with the effects of CoQ10. Knockdown of Coa6 abolished the beneficial effects of CoQ10 in this model.

## Materials and methods

### Network pharmacology analysis

We initially performed target gene prediction by searching the DrugBank database (https://go.drugbank.com/drugs/) using keyword “Ubidecarenone” and the Comparative Toxicogenomics Database (https://ctdbase.org/) using keyword “Ubiquinone”. In parallel, the MitoCarta 3.0 database (https://www.broadinstitute.org/mitocarta/mitocarta30-inventory-mammalian-mitochondrial-proteins-and-pathways) was searched for mouse mitochondria-related genes. Through this process, a total of 162 CoQ10-related genes and 1140 mitochondria-related genes in mice were obtained. Subsequently, the 162 CoQ10-associated genes were intersected with the 1140 mitochondrial genes, resulting in the identification of 30 common target genes. Protein–protein interaction analysis was then performed to explore the interactions among the CoQ10-related mitochondrial genes using the STRING database (https://cn.string-db.org/) and visualized with the Cytoscape software version 3.9.1, with genes ranked according to their interaction degree. Thereafter, Gene Ontology (GO) and Kyoto Encyclopedia of Genes and Genomes (KEGG) pathway enrichment analyses of the key targets were conducted using the DAVID database and the Microbiotics platform. GO enrichment analyses were performed for cellular components (GO-CC), biological processes (GO-BP), and molecular functions (GO-MF). KEGG pathway enrichment results were visualized using Chiplot software (https://www.chiplot.online/) to identify potential pathways involved in the regulation of mitochondrial functions associated with neurodegenerative pathologies. Finally, target gene prediction and validation were further assessed using the Gene Set Enrichment Analysis (GSEA) database (https://www.gsea-msigdb.org/gsea/index.jsp).

### Animals

We purchased four types of transgenic mice: (1) Pcp2^Cre^ mice with specific insertion of Cre enzyme into all PCs (Strain #: 004146, Jackson Laboratories, Bar Harbor, ME) [22]; (2) Drp1^*fl/fl*^ mice with insertion of loxP sites flanking the *Drp1* sequence (S-CKO-15806, Cyagen, Guangzhou, China) [23]; (3) B6/JGpt-H11^em1Cin(CAG-LoxP-ZsGreen-Stop-LoxP-tdTomato)^/Gpt mice (B6-G/R mice) with insertion of loxP sites flanking the ZsGreen sequence (Strain #: T006163, GemPharmatech, Nanjing, China); and (4) RoSA26-CAG-LSL-GFP-MITO-labeled mice (Mito-GFP mice) with insertion of loxP sites flanking the green fluorescent protein (GFP) specifically located on the mitochondrial outer membrane (GemPharmatech). All mice were housed (up to five per cage) under a 12-h light–dark cycle (from 8:00 a.m. to 8:00 p.m.) with *ad libitum* access to food and water. All animal experiments were performed in accordance with the Guidelines for the Care and Use of Laboratory Animals and were approved by the Animal Care and Use Committee of the Air Force Military Medical University (Approval No. IACUC-20190107).

### Generation of PC-Drp1^−/−^ mice, PC^tdTomato^ mice, PC^tdTomato^-Drp1^−/−^ mice, PC^Mito^ mice, and PC^Mito^-Drp1^−/−^ mice

The PC-Drp1^−/−^ mice were generated by crossing Pcp2^Cre^ mice with Drp1^*fl/fl*^ mice, in which *Drp1* gene was specifically knocked out in cerebellar PCs. The PC^tdTomato^ mice were generated by crossing Pcp2^Cre^ mice with B6-G/R mice, in which Pcp2-positive PCs carry tdTomato. The PC^tdTomato^-Drp1^−/−^ mice were generated by crossing Drp1^*fl/fl*^ mice with B6-G/R mice, in which the specific Drp1-deficiency PCs expressed tdTomato. The PC^Mito−GFP^ mice were generated by crossing Pcp2^Cre^ mice with Mito-GFP mice, in which Pcp2-positive PCs carry GFP. The PC^Mito^-Drp1^−/−^ mice were generated by crossing Drp1^*fl/fl*^ mice with Mito-GFP mice, in which mitochondrial outer membrane within the specific Drp1-deficiency PCs express GFP.

### Identification of transgenic mice via PCR analysis

Each mouse tail sample was lysed in a mix of 200 µL Lysis buffer and 4 µL DNA release solution. The mixture was boiled at 55 °C for 30 min and 98 °C for 5 min, then centrifuged at 12,000 rpm for 5 min. The obtained supernatant containing DNA sample was temporarily stored at 4 °C for subsequent experiments. The mouse tail identification kit (TSE014, Tsingke, Xi’an, China) was used for DNA PCR and primer sequences are listed in Table S1. The PCR protocol included pre-denaturation at 94 °C for 3 min, followed by 35 cycles of denaturation at 94 °C for 30 s, annealing at 60 °C for 35 s, and finally extended at 72 °C for 35 s. PCR products were analyzed by agarose gel electrophoresis to determine the genotype.

### Rotarod test

For motor coordination assessment, the rotarod test (BZY007, Jiliang, Shanghai, China) started at 4 rpm and increased to 40 rpm within 180 s. Each trial lasted 5 min, with at least a 15-min interval. Mice were trained 3 times daily for 3 consecutive days, and data were collected on the fourth day. The latency to fall from the rod was recorded.

### Open field test

To evaluate the free and spontaneous movements of transgenic mice, the animals were placed in a 50 × 50 × 50 cm^3^ opaque square box (DigBehav, Shanghai, China). The light was uniformly diffused by the top LEDs (100 lx), and their movements were recorded for 15 min using a camera connected to a computer. Mice were acclimated to the environment for 30 min before the test. Mouse movements were automatically tracked, and the total distance traveled was analyzed using dedicated software.

### Morris water maze test

The long-term spatial memory of PC-Drp1^−/−^ mice was evaluated with a Morris water maze apparatus (TECHMAN, Shenzhen, China). At least five mice were included in each group. The maze was divided into four quadrants based on the position of a marker. In the training phase, a platform was placed in the center of the first quadrant, and each mouse was introduced into the pool at a random point, facing the pool wall. A video recording system was used to capture the latency to locate the platform (escape latency) and their swimming paths. The mice were placed in the water at different starting points (from different quadrants) in each trial. If the mice failed to locate the platform within 60 s, they would be guided to the platform and stayed there for 15 s. Each mouse performed four trials daily, with an inter-trial interval of at least 15 min. The average escape latency across the four trials was recorded as the learning performance for that day. Over five consecutive days of training, the swimming speed (mm/s) and escape latency (s) were measured.

### Eight-arm maze test

The working memory of PC-Drp1^−/−^ mice was assessed by an eight-arm radial maze test (TECHMAN). At least five mice were included in each group. Animals were acclimated to the experimental environment for one week, during which they were weighed and fasted for 24 h before the experiment. Following fasting, normal food (2–3 g) was provided daily at the end of training sessions to maintain the body weight at 80%–85% of their normal feeding weight. On days 1 and 2, food pellets were scattered across each arm and the central area of the maze to familiarize the animals with the setup. On day 3, individual training began, where a single food pellet was placed at the end of each arm near the outer food box, allowing the mice to explore and eat freely. From days 4 to 7, the food was placed inside the boxes, and training procedure was repeated as that on day 3. Working memory performance was assessed from day 3 to day 7. If a mouse re-entered an arm that had been visited before during the same experiment, a working memory error was considered. This behavior indicates that the mice failed to maintain and update the information regarding the "visited vs. unvisited" arm paths, which is a widely accepted measure of working memory in classic literature [24]. Additionally, the search strategies of mice were manually categorized based on angles of 45°, 90°, 135°, and 180°. Over a period of five consecutive days, we assessed the working memory performance of mice by measuring working memory errors and the percentages of searching strategies categorized at angles of 45°, 90°, 135°, and 180°.

### Golgi staining

Golgi staining was performed using a Golgi staining kit (PK401, FD NeuroTechnologies, Columbia, MD). After deep anesthesia (pentobarbital sodium, 40 mg/kg, intra-peritonelly), brain tissue was quickly extracted, and blood on the surface of the tissue was rinsed off with distilled water. The tissue was immersed in a mixture of solution A and solution B. On the following day, the immersion solution was replaced with a fresh solution, and the tissue was kept in the dark at room temperature for two weeks. Subsequently, the tissue was transferred to solution C and kept in the dark at room temperature for at least 72 h, with the solution changed at least once on the second day. The tissue was then sectioned into 100-μm-thick slices at a temperature of − 20 °C to − 22 °C using a cryotome and transferred onto slides coated with 3.5% gelatin. The slides were air-dried in the dark at room temperature for three days. The sections were washed twice with double-distilled water for 4 min each. Then they were incubated in a mixture of 1 part solution D, 1 part solution E, and 2 parts double-distilled water for 10 min, followed by rinsing with distilled water for 2 × 4 min. The sections were dehydrated through an ethanol series (50%, 75%, and 95% ethanol, each for 4 min) and then dehydrated in absolute ethanol four times, 4 min each. Subsequently, the sections were cleared in xylene for 3 × 4 min. Finally, the coverslips were mounted using resin sealant. PCs were observed under an optical microscope (CX33, Olympus, Tokyo, Japan), and Sholl analysis was performed using ImageJ software [25], focusing on two parameters: the abundance of PCs and the density of their dendritic spines.

PC morphology, including dendritic complexity and spine density, was analyzed. Dendritic complexity was assessed via Sholl analysis [26], where the number of dendritic intersections with concentric circles (with a radius interval of 40 µm) was quantified. Separately, spine density was measured by manually counting all visible spines on a defined distal dendritic segment and normalizing to its length (spines/µm). For each experimental condition, Sholl analysis included at least 6 PCs, and spine density was measured on at least 12 dendrites, all derived from 3 biological replicates.

### Immunofluorescence staining

All mice were deeply anesthetized with 40 mg/kg pentobarbital sodium (Merck, Darmstadt, Germany), followed by transcardiac perfusion of 4% paraformaldehyde. The extracted brains were fixed in 4% paraformaldehyde (R20497-10, Source Leaf) for 24 h and then immersed in 30% sucrose solution for 2 days. Brain sections were cut into 30-μm-thick slices using a cryostat (Leica CM1850, Wetzlar, Germany) and mounted onto slides. The sections were blocked for 30 min with a blocking solution containing 0.3% Triton X-100, 10% calf serum, and PBS, and then incubated overnight at 4 °C with the following primary antibodies: rabbit anti-Drp1 (1:50, Ab184247, Abcam), mouse anti-postsynaptic density protein 95 (PSD95)(1:500, MA1-045, Invitrogen), rabbit anti-Coa6 (1:200, 24209-1-AP, Proteintech, Wuhan, China) and rabbit anti-IBA1 (1:500, 011-27991, Wako, Tokyo, Japan). After three washes with PBS, the sections were incubated for 2 h with secondary antibodies, including DyLight 647 goat anti-mouse IgG (1:5000, A23610, Abbkine, Wuhan, China) or DyLight 647 goat anti-rabbit IgG (1:5000, A23620, Abbkine). The sections were washed three times with PBS, and cell nuclei were stained with DAPI (1:2500, C1002, Beyotime, Shanghai, China) for 5 min. After air drying, the slides were sealed with a fluorescent mounting medium and observed using a confocal laser scanning microscope (Leica STELLARIS5). ImageJ software was used for statistical analysis.

### Multiple immunohistochemical staining

Multiple immunohistochemical staining was performed using a Tyramide Signal Amplification kit (Abclonal, RK50025P, Wuhan, China) on cerebellar cryosections. Briefly, thawed sections were washed 3 × 5 min in TBST, permeabilized with 0.3% Triton X-100 at room temperature (RT) for 20 min, and blocked for peroxidase activity in hydrogen peroxide (room temperature, 15 min, dark) and for non-specific binding in 3% bovine serum albumin (room temperature, 30 min). They were then incubated sequentially with a primary antibody (37 °C, 1.5 h), an HRP-conjugated secondary antibody (room temperature, 50 min, dark), and fluorescent dye (1:200 in TSA solution, room temperature, 5 min, dark). For staining of multiple targets, the antibody complex was eluted using a specific buffer (37 °C, 2 × 10-min, in the dark; Abclonal, RM02984P), and the blocking-incubation-amplification cycle was repeated for each subsequent primary antibody. The following primary antibodies were used: rabbit anti-Calbindin (1:2500, ab229915, Abcam), rabbit anti-Drp1 (1:500, ab184247, Abcam), rabbit anti-Coa6 (1:1000, 24209-1-AP, Proteintech), rabbit anti-GFAP (1:2500, GB11096, Servicebio, Wuhan, China), rabbit anti-IBA1(1:1000, ab178847, Abcam), and rabbit anti-NeuN (1:1000, ab177487, Abcam). Finally, nuclei were counterstained with DAPI (room temperature, 10 min, dark), and sections were mounted with an anti-fade medium.

### Mitochondrial network analysis (MiNA)

Mitochondrial networks were analyzed from maximum intensity projections of confocal z-stacks of cerebellar sections. Image preprocessing in ImageJ consisted of conversion to 8-bit, application of Unsharp Mask and CLAHE for contrast enhancement, and Median filter denoising. Mitochondrial network structure was quantified by converting preprocessed images to binary format, performing skeletonization, and analyzing the skeletons using the built-in "Analyze Skeleton" tool and the MiNA plugin. The quantified parameters included: Number of Individuals, Number of Networks, Branch Lengths, and Mean Branches per Network [27]. Analysis was conducted on a minimum of 6 images per condition from 3 biological replicates, as per standard practice.

### Transmission electron microscopy analysis

Three mice from each group (Control, PC-Drp1^−/−^, and CoQ10 intervention groups) were selected for perfusion sampling. The cerebellar tissues were placed in 2.5% glutaraldehyde at 4 °C overnight and washed three times with PBS. The tissues were then fixed in 1% osmium tetroxide at 4 °C in the dark for 2 h and washed three times with PBS. Subsequently, the samples were dehydrated through an ethanol gradient (15 min per step) and finally dehydrated in 100% acetone before being embedded in resin. Eleven 70-nm sections were collected onto copper grids and stained with lead citrate and uranyl acetate for 10 min. Images were captured using a transmission electron microscope (JEM1400, JEOL, Tokyo, Japan).

Transmission electron microscopy images were analyzed using ImageJ. The scale was calibrated for each image using the "Set Scale" function to ensure all measurements reflected actual dimensions. Mitochondrial morphology was assessed by manually tracing the outer membrane of individual mitochondria to obtain the area, perimeter, and circularity (calculated as 4π × Area/Perimeter^2^). To quantify cristae density, the total area of cristae within each mitochondrion was determined by manually outlining all discernible cristae folds. The cristae area ratio (cristae density) was then calculated as the total cristae area divided by the corresponding mitochondrial area. For each experimental condition, analyses were performed on at least *n* = 3 biological replicates, as commonly employed in similar ultrastructural studies [28, 29].

### Mitochondria isolation

Mitochondria isolation was performed using the mitochondrial extraction kit according to the instructions (SM0020, Solarbio, Beijing, China). Cerebellum tissues in different groups were rinsed in saline and homogenized in Tris–HCl-based lysis buffer. The homogenization was centrifuged at 1000× *g* for 5 min at 4 °C. The supernatant was transferred to a new tube and centrifuged under the same conditions. The secondary supernatant was then centrifuged at 12,000× *g* for 10 min at 4 °C. The pellet was resuspended in Tris–HCl-based wash buffer and centrifuged at 1000× *g* for 5 min at 4 °C. The supernatant was centrifuged at 12,000× *g* for another 10 min at 4 °C. Finally, the mitochondria were resuspended in phosphate-based store buffer (pH 7.2–7.4).

### Mitochondrial membrane potential (MMP) assay

In accordance with the protocol provided by the Mitochondrial JC-1 Assay Kit (C2005, Beyotime), the concentrations of the isolated mitochondria were adjusted to a standardized level, followed by staining with the JC-1 dye. Fluorescence intensity measurements were obtained using a fluorescence microplate reader (Spark TECAN, Männedorf, Switzerland) at excitation wavelengths ranging from 475 to 520 nm.

### Reactive oxygen species (ROS) detection

Cerebellar tissues were digested to make a cell suspension. ROS levels in the collected cells were determined using the Mitochondrial ROS Assay Kit (S0033S, Beyotime), according to the manufacturer’s instructions. Cells loaded with probes were detected by flow cytometry. The maximum excitation wavelength in flow cytometry was 488 nm and the maximum emission wavelength was 525 nm (B75442, Beckman Coulter, Brea, CA). FlowJo 10.8.1 software was used for analysis.

### Adenosine triphosphate (ATP) detection

ATP levels were measured using an Enhanced ATP Assay Kit (S0027, Beyotime), following the manufacturer's protocol. Cerebellar tissues were homogenized in lysis buffer and centrifuged (12,000× *g*, 5 min, 4 °C) to collect the supernatant. A standard curve was prepared by serial dilution (0.01, 0.03, 0.1, 0.3, 1, 3, and 10 µM). In a black 96-well plate, 100 µL of detection reagent was added per well and allowed to stabilize for 3 min. After adding 20 µL of sample or the standard, the plate was immediately mixed, and luminescence was recorded using a microplate reader (Spark TECAN). The ATP concentrations were calculated based on the standard curve, and normalized to the total protein concentration and expressed as nmol per mg of protein (nmol/mg).

### Mitochondrial metabolism assays

Cerebellar tissues were digested with 0.25% trypsin. The reaction was terminated with serum-containing medium, and digest filtered through a 70-μm strainer. Cells were pelleted by centrifugation at 300× *g* for 5 min.

For oxygen consumption rate assay (E-BC-F070, Elabscience, Wuhan, China), we resuspended cells in probe working solution, plated 100 µL per well in a black plate, and incubated at 37 °C for 30 min. After adding 80 µL of sealing solution to each well, fluorescence was measured kinetically from the bottom of the plate at 37 °C (Ex/Em: 405/650 nm), with readings taken every 3 min for 90 min (Spark TECAN).

For extracellular acidification rate (ECAR) detection (E-BC-F069, Elabscience), the cells were washed in culture medium (500 ×  *g*, 5 min), and finally resuspended in 200 μL of the probe working solution. For the assay, 150 μL of the cell suspension was seeded into a black 96-well plate. The ECAR was determined by kinetically measuring fluorescence (490/535 nm) every 3 min for 120 min using a microplate reader (Spark TECAN).

For fatty acid oxidation detection (E-BC-K784-M, Elabscience), the pelleted cells were washed three times with saline (200 μL each) and then homogenized in 200 μL of saline. After centrifugation (10,000× *g*, 15 min, 4 °C), the supernatant was collected for assay and protein concentration determination. For the reaction, 50 μL of sample was mixed with 165 μL of substrate, 145 μL of reaction working solution, and 20 μL of chromogenic agent in sequence in a well. The plate was shaken, incubated at 37 °C for 30 min, and read at 450 nm (Spark TECAN).

### Western blot

Cerebellar tissue was homogenized in pre-cooled RIPA lysis buffer (P0013B, Beyotime), and cells were lysed using a pre-cooled lysis buffer (R0010, Solarbio, Beijing, China). The lysates were centrifuged at 12,000 rpm for 10 min at 4 °C, and the supernatant was collected. Protein concentration was measured using a BCA kit (ZJ102L, EpiZyme, Shanghai, China). Proteins were separated by 12.5% SDS-PAGE (PG113, Yadase) and transferred onto a 0.45-µm PVDF membrane (Millipore, Burlington, MA). The membranes were blocked in a rapid protein-free blocking solution (PS108P, Yadase) for 20 min. The primary antibodies used included rabbit anti-DRP1 polyclonal antibody (1:1000, Ab184247, Abcam), rabbit anti-superoxide dismutase 1 (SOD1) polyclonal antibody (1:1000, A0274, ABclonal), rabbit anti-glutathione peroxidase 1 (GPx1) polyclonal antibody (1:1000, A11166, ABclonal), mouse anti-Total OXPHOS polyclonal antibody (1:300, Ab110413, Abcam), cytochrome *c* oxidase subunit IV (COX4) antibody (1:1000, PA5-29992, Invitrogen), rabbit anti-Coa6 (1:1000, 24209-1-AP, Proteintech) and rabbit anti-beta actin polyclonal antibody (1:5000, AC004, ABclonal). The PVDF membranes were incubated with the primary antibodies overnight at 4 °C and washed three times with TBST. Secondary antibodies included HRP-conjugated goat anti-mouse IgG (1:5000, A21010, Abbkine) and HRP-conjugated goat anti-rabbit IgG (1:5000, A21020, Abbkine). Protein bands were visualized using Fusion FX EDGE chemiluminescence imaging technology, and the gray values of the bands were quantified using ImageJ software.

### Quantitative real-time PCR (qPCR)

Following total RNA extraction with Trizol reagent (DP419, TIANGEN), cDNA was synthesized using a reverse transcription kit (AG11728, Accurate Biology, Zhejiang, China). qPCR was conducted using SYBR Green master mix (AG11701, Accurate Biology) on a real-time PCR system. *Gapdh* was used as the reference gene. The PCR protocol comprised an initial step at 95 °C for 30 s and 40 cycles of 95 °C for 5 s and 60 °C for 30 s. Amplification specificity was verified by melting curve analysis. Gene expression was quantified via the 2^−ΔΔCt^ method. The primer sequences used for *Coa6* amplification were as follows: Forward: 5′-CATGAAGGAAAGGCAGGCATG-3′; Reverse: 5′-TGAAAGTCCAGAAAGAGCCTAGG-3′.

### Long-term supplementation with CoQ10 in mice

PC-Drp1^−/−^ mice, PC^tdTomato^-Drp1^−/−^ mice, and PC^Mito^-Drp1^−/−^ mice received either CoQ10 supplementation or drinking water containing 3% dimethyl sulfoxide (DMSO; vehicle) from postnatal day 15 to day 90. CoQ10 (HY-N0111, MCE, NJ) was dissolved in a 3% DMSO solution at a concentration of 500 µM. The CoQ10 solution was added to the drinking water and refreshed daily, which is consistent with previous research [30, 31].

### Thermal proteome profiling

First, the mouse brain tissue was lysed by a lysis buffer to obtain the protein solution. After high-speed centrifugation of the tissue lysate, the supernatant was collected and passed through an ultrafiltration tube with PBS three times. After determining the protein concentration by the BCA method, the supernatant (protein concentration of 2 mg/mL) was treated with 10 μM CoQ10, 100 μM CoQ10 or an equal volume of DMSO (three replicates for each treatment) at 4 °C for 2 h. Samples were then heated at 58 °C for 7 min and centrifuged at 4 °C for 30 min at 22,000× *g*. The supernatant was transferred to a new EP tube, after which pre-cooled acetone was added to precipitate the protein at − 80 °C. Next, proteomic identification and analysis were performed by Orbitrap HF-X LC–MS/MS high-resolution mass spectrometry. HFX was used for mass spectrometry signal acquisition (with a 70-min time gradient for each sample). Data analysis was conducted using MaxQuant analysis software. A search on the database of mouse proteins annotated with Swiss-Prot in UniProt yielded approximately 3100 proteins identified in total. Differential analysis of the omics data had two priorities. The first-priority cutoff criterion was proteins with significantly different levels between the 10 μM CoQ10 and the DMSO group as a presence-versus-absence distinction, and the levels increased with rising drug concentration. The second-priority cutoff was proteins with levels showing a presence-versus-absence difference compared to the DMSO group, but not increasing in the high-concentration group.

### Plasmid construction and protein purification

From the first-priority screening data, the Coa6 protein was selected as the candidate target protein for validation. First, RNA was extracted from mouse macrophages, and cDNA was generated through reverse transcription. Then, PCR was performed to amplify the *Coa6* gene. The coding sequence of *Coa6* was cloned into the prokaryotic expression vector PET28A by homologous recombination to generate PET28A-Coa6. After sequencing, the plasmid was transformed into BL21 (DE3) competent cells, and the bacterial culture was then transferred to a 1-L conical flask. When the OD of the bacterial solution reached ~ 0.8, 1 mM of IPTG inducer was added and induced for 12 h at 37 °C. Then proteins were extracted from the bacteria by ultrasonic lysis, and protein supernatant was purified by Ni-beads. The impurity proteins were eluted successively through 20 mM imidazole, 50 mM imidazole, and 500 mM imidazole, and the target proteins were eluted. Finally, the 500 mM imidazole elution solution was subjected to SDS-PAGE staining to determine the protein purity.

### Cell thermal shift assay (CETSA)

The Coa6 protein solution was added to separate tubes containing increasing concentrations of CoQ10 (dissolved in DMSO vehicle), respectively. To each 100 μL reaction volume, CoQ10 was added at final concentrations of 0 μM (DMSO control), 0.32 μM, 1.6 μM, 8 μM, 40 μM, and 200 μM. After 1.5-h incubation at room temperature, samples were heated at 55 °C for 7 min in a water bath, followed by high-speed centrifugation (4 °C, 20,000× *g*, 30 min). Subsequently, the supernatant was collected for further SDS-PAGE. The electrophoretic results showed that the band intensity of the target protein increased with the increasing drug concentrations.

### Tissue CETSA

Mouse cerebella were rapidly dissected and homogenized in a cryolysis buffer containing protease inhibitors. The tissue lysate was centrifuged at 12,000× *g* for 10 min at 4 °C, and the protein concentration was determined by the BCA method. Protein concentrations were adjusted to be identical across the samples before heat treatment. Equal amounts of cerebellar proteins were treated with a preheated hot block at temperature gradients (42, 45, 49, 53, 56, 60 and 65 °C) for 5 min, and then immediately cooled on ice for 5 min to allow the heat-denatured proteins to aggregate. The samples were then centrifuged at 12,000 × *g* for 10 min at 4 °C to form insoluble protein aggregates. The supernatant containing the soluble protein portion was collected and analyzed by Western blotting using the anti-Coa6 antibody. The relative thermal stability of Coa6 under different experimental conditions was qualitatively analyzed.

### Drug affinity responsive target stability (DARTS)

The Coa6 protein solution was mixed with varying concentrations of CoQ10. Specifically, 100 μL of the solution was supplemented with CoQ10 at concentrations of 0 μM (DMSO control), 1 μM, 10 μM, 100 μM, and 200 μM, respectively. After incubating for 1.5 h at room temperature, each sample was treated with pronase E at 1:100 mass ratio and incubated in a 37 °C water bath for 15 min. Following the reaction, the samples were immediately prepared for SDS-loading by boiling, and the supernatants were subjected to SDS-PAGE. As the CoQ10 concentration increased, the Coa6 protein binding to it showed enhanced resistance to enzymatic degradation.

### Surface plasmon resonance

The CM5 sensor chip activator was prepared by mixing 400 mM EDC and 100 mM NHS immediately prior to injection. The CM5 sensor chip was activated for 420 s with the mixture at a flow rate of 10 μL/min. Coa6 was diluted to 20 μg/mL in the immobilization buffer, then injected to sample channel Fc2 at a flow rate of 10 μL/min, typically resulting in immobilization levels of 12,600 RU. The reference channel Fc1 did not need the ligand immobilization step. The chip was deactivated by 1 M ethanolamine hydrochloride at a flow rate of 10 μL/min for 420 s. CoQ10 was diluted with the same analyte buffer to 8 concentrations (0.39–25 μM). CoQ10 was injected to channels Fc1–Fc2 at a flow rate of 20 μL/min for an association phase of 100 s, followed by 180 s of dissociation. The association and dissociation processes were handled in the analyte buffer. This cycle in an ascending order of the analyte concentration was repeated 8 times. After each cycle of interaction analysis, the chip was regenerated.

### Molecular docking of Coa6 with CoQ10

Molecular docking of Coa6 with CoQ10 was analyzed based on previous descriptions [32]. The structure of the Coa6–CoQ10 complex was predicted utilizing the ProtENIX web (https://proteiniq.io/app/protenix) server under default parameters and subsequently visualized through the academic edition of Maestro. The three-dimensional architectures of the proteins were rendered and explored using ChimeraX (https://www.rbvi.ucsf.edu/chimerax/).

### ELISA for CoQ10

CoQ10 levels in serum and cerebellar supernatants (homogenized in PBS and centrifuged at 5000 × *g*) were determined using an ELISA kit (F30469-A, FANKEW, Shanghai, China). Briefly, 10 µL samples were added to wells with 40 µL diluent and incubated at 37 °C for 30 min. After five-step washing, the enzyme conjugate was added. Following a second wash, 50 µL of substrates A and B were added sequentially to each well (10 min, 37 °C, dark). The reaction was stopped, and absorbance was read at 450 nm after 15 min (Spark TECAN).

### Metabolic cage experiments

Mice were individually housed and monitored for 3 days in metabolic cages within a controlled environment (12-h light/dark cycle). Water intake, oxygen consumption (VO₂), and carbon dioxide production (VCO₂) were automatically recorded every 40 min. The respiratory exchange ratio (RER) was calculated as follows: RER = VCO₂/VO₂. Energy expenditure (EE) was calculated as follows: EE = (3.941 × VO₂ + 1.106 × VCO₂) × 1.44 (unit: Kcal/h).

### Patch-clamp electrophysiologic recording

Whole-cell electrophysiological experiments were conducted in control and PC-Drp1^−/−^ mice. The mice were anesthetized by intraperitoneal injection of pentobarbital sodium (40 mg/kg) and perfused with 20 mL of an ice-cold carbogenated cutting solution (95% O₂, 5% CO₂), which contained 240 mM sucrose, 2.5 mM KCl, 1.25 mM Na₂HPO₄, 2 mM MgSO₄, 1 mM CaCl₂, 26 mM NaHCO₃, and 10 mM *D*-glucose. The brain was then quickly removed and placed in the same cold, carbonated cutting solution to prepare for slicing. Sagittal brain slices (250-μm thick) were prepared using a microtome (Leica VT1200S) and incubated in the cutting solution in a holding chamber at 32 °C for 30 min. The slices were subsequently transferred to artificial cerebrospinal fluid containing (in mM): 119 NaCl, 2.3 KCl, 1.0 NaH_2_PO_4_, 26 NaHCO_3_, 11 D-(+)-glucose, 1.3 MgCl_2_·6H_2_O, and 2.5 CaCl_2_·2H_2_O (pH 7.4; osmotic pressure 295–300 mOsm/L) and kept at room temperature for at least 1 h. CoQ10 was added to the artificial cerebrospinal fluid at a final concentration of 11 μM. Slices from 3 mice were incubated under these conditions. The sections were placed in a recording chamber maintained at 24–28 °C (TC-324B, Warner Instrument, Hamden, CT) with a perfusion rate of 2.0 mL/min. Whole-cell patch-clamp recordings were performed under infrared differential interference contrast (IR-DIC) visual guidance. Recording pipettes (BF150-86-7.5, Sutter Instrument, Novato, CA) were fabricated using a horizontal pipette puller (P-97, Sutter Instrument) and had a tip resistance of 3–6 MΩ. The patch pipettes were filled with an intracellular solution containing (in mM): 128 potassium gluconate, 10 HEPES, 10 sodium creatine phosphate, 1.1 EGTA, 5 magnesium ATP, and 0.4 GTP sodium. pH was adjusted to 7.3 with potassium hydroxide, and the osmotic pressure was adjusted to 300–305 mOsm using sucrose. During recordings, cells with a series resistance greater than 20 MΩ were excluded from further analysis. Neurons with a resting membrane potential more negative than − 60 mV and capable of firing action potentials were selected for subsequent experiments. Liquid junction potentials were not corrected. Currents or membrane potentials were recorded using an Axon 200A amplifier (Molecular Devices, Sunnyvale, CA). The signals were low-pass filtered at 5 kHz and digitized at 20 kHz using a Digidata 1322A system and Clampex 9.0 software (Molecular Devices). The data were stored on a computer for subsequent offline analysis [33].

### ZD7288 intervention

Following anesthesia, mice were secured in a stereotaxic apparatus. A burr hole was drilled at the target coordinates (6.25 mm posterior to bregma, ± 0.5 mm lateral to the midline), and two anchor screws were implanted in surrounding non-functional areas. A sterile guide cannula was then implanted vertically, with its tip positioned 0.5 mm above the target cerebellar region. The cannula pedestal, anchor screws, and exposed skull were permanently secured with dental cement. After a 3-day recovery period, mice underwent eight-arm maze training for 6 days. On day 7, a baseline behavioral test was performed (pre-treatment). Mice were then briefly re-anesthetized and restrained, and ZD7288 (1 µg/site in saline; HY-101346, MCE) was infused into the cerebellum via an internal cannula at a rate of 0.1 µL/min. The internal cannula was left in place for 5 min post-infusion. Behavioral testing was repeated 1 h after the infusion.

### Viral injection

Following anesthesia, mice were secured in a stereotaxic apparatus (RWD 69100). Adeno-associated viruses (AAVs) were diluted to 1 × 10^12^ vg/mL and infused into the cerebellum using a microsyringe (RWD 79013) at a rate of 100 nL/min. The injection needle was left in place for 8 min post-infusion before withdrawal.

For Coa6 down-regulation, pAAV-CMV-DIO-mCherry-miR30shRNA(Coa6)-WPRE (Y43119, OBiO) and its control pAAV-CMV-DIO-mCherry miR30shRNA(NC)-WPRE (Y33435, OBiO) were used. For Coa6 overexpression, pAAV-CMV-DIO-Coa6-3xFLAG-P2A-mCherry-WPRE (H148026, OBiO) and its control pAAV-CMV-DIO-mCherry-WPRE (H33813, OBiO) were used. Viruses were bilaterally injected at the following sites: AP ± 0.50 mm, ML – 6.25 mm, DV – 2.25 mm (1 µL) and AP ± 0.50 mm, ML − 7.00 mm, DV − 3.00 mm (1 µL). Behavioral, morphological, and functional analyses were conducted three weeks post-injection.

### Statistical methods

All statistical analyses were performed using GraphPad Prism 8. For comparisons between two groups, *t*-test was used for parametric data, while the Mann–Whitney *U* test was applied for non-parametric data. For multiple comparisons, one-way ANOVA followed by Dunnett’s *t* test was used for parametric data, and the Kruskal–Wallis test was employed for non-parametric data. For repeated measures data, two-way ANOVA followed by the Sidak test was used. A *P* value of less than 0.05 was considered statistically significant. Data are presented as mean ± SD. For statistical analysis, technical replicates were first averaged, and only the resulting mean value for each biological replicate was used as an independent *n* for statistical testing. The numbers of mice are provided in the corresponding figure legends. The statistical methods employed in this study have all been independently reviewed by professional statisticians, and it has been confirmed that the selection of statistical methods and the interpretation of results comply with the prescribed requirements.

## Results

### CoQ10 is linked to the *Dnm1l*/*Drp1* gene

**Fig. 1:**
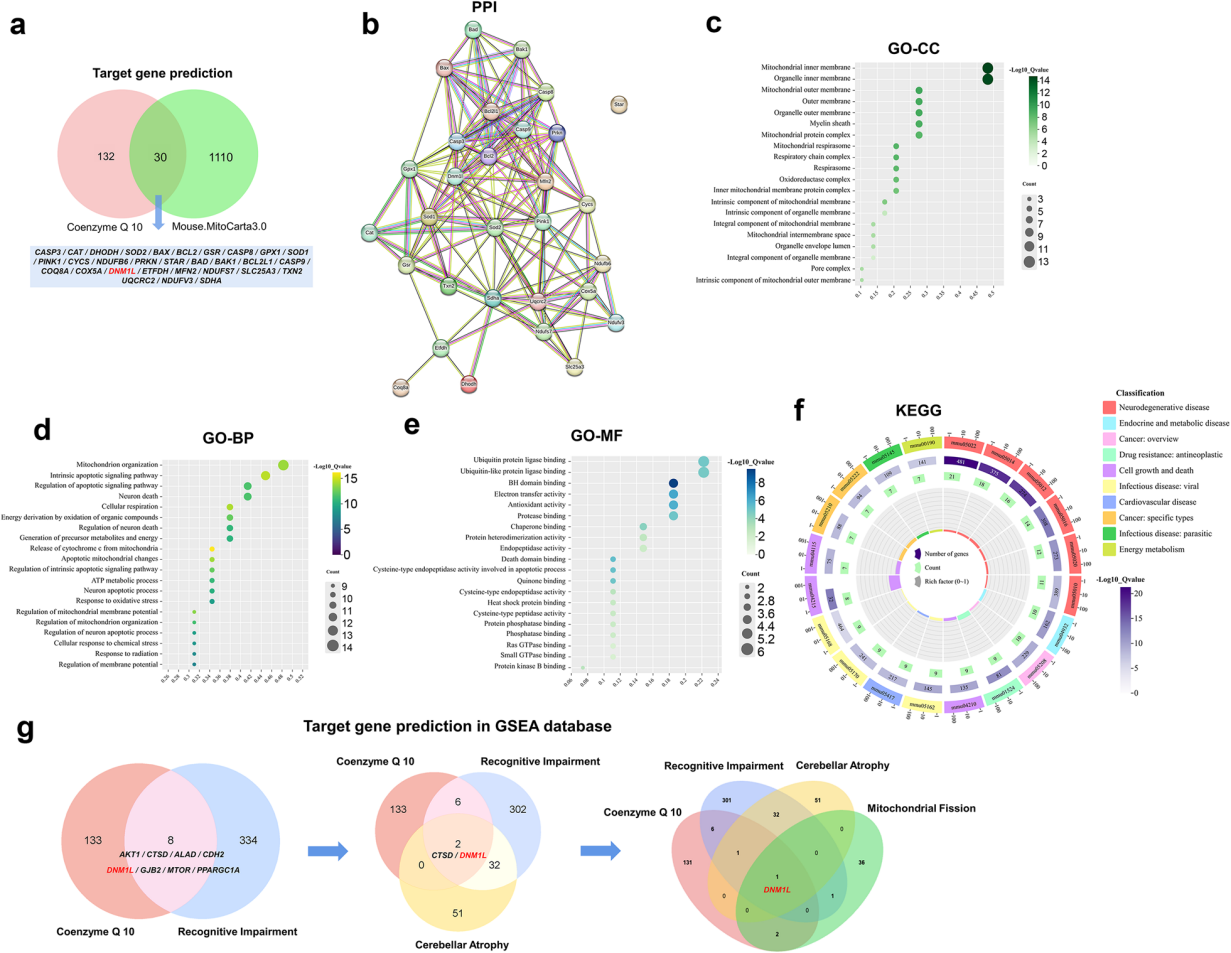
CoQ10 is associated with the *dnm1l* gene. **a-g** Network pharmacology analysis composed of target gene prediction (**a**), protein–protein interaction (PPI) (**b**), Gene Ontology-cellular components (GO-CC) (**c**), Gene Ontology-biological processes (GO-BP) (**d**), Gene Ontology-molecular function (GO-MF) (**e**), Kyoto Encyclopedia of Genes and Genomes (KEGG) (**f**) and target gene prediction in GSEA database (**g**). Network pharmacology analysis elucidated the correlation between Drp1 deficiency and CoQ10 supplementation

Network pharmacology analysis (Fig. S1) identified a total of 30 target genes of CoQ10 based on CoQ10-related gene databases which contain 162 genes, and a mitochondria-related gene database which contains 1140 genes (Fig. 1a). Protein–protein interaction analysis of these 30 genes revealed that *Sod2* (superoxide dismutase 2), *Caspase-3*, *Sdha* (succinate dehydrogenase complex subunit A), and *Dnm1l* (also known as *Drp1*) were the most significant nodes within the network (Fig. 1b). The GO-CC analysis revealed significantly enriched cellular components including the inner and outer mitochondrial membranes, as well as organelle membranes (Fig. 1c). The GO-BP analysis revealed enriched biological processes including mitochondrial respiration and apoptotic signaling (Fig. 1d). The GO-MF analysis revealed enriched molecular functions including ubiquitin protein ligase binding, ubiquitin-like protein ligase binding, and BH domain binding (Fig. 1e). KEGG analysis illustrated that the enriched genes were mainly associated with neurodegenerative diseases, such as AD, PD, and HD (Fig. 1f).

A further target gene prediction showed that the *Dnm1l*/*Drp1* gene was the most important gene, linked to all four aspects: CoQ10, cognitive impairment, cerebellar atrophy, and mitochondrial division (Fig. 1g). The above results suggest that CoQ10 is associated with the *Dnm1l*/*Drp1* gene in cognitive impairment caused by cerebellar atrophy. Thus, CoQ10 may have therapeutic potential for Drp1-related disorders.

### Drp1 deficiency in PCs causes working memory defects in mice

**Fig. 2:**
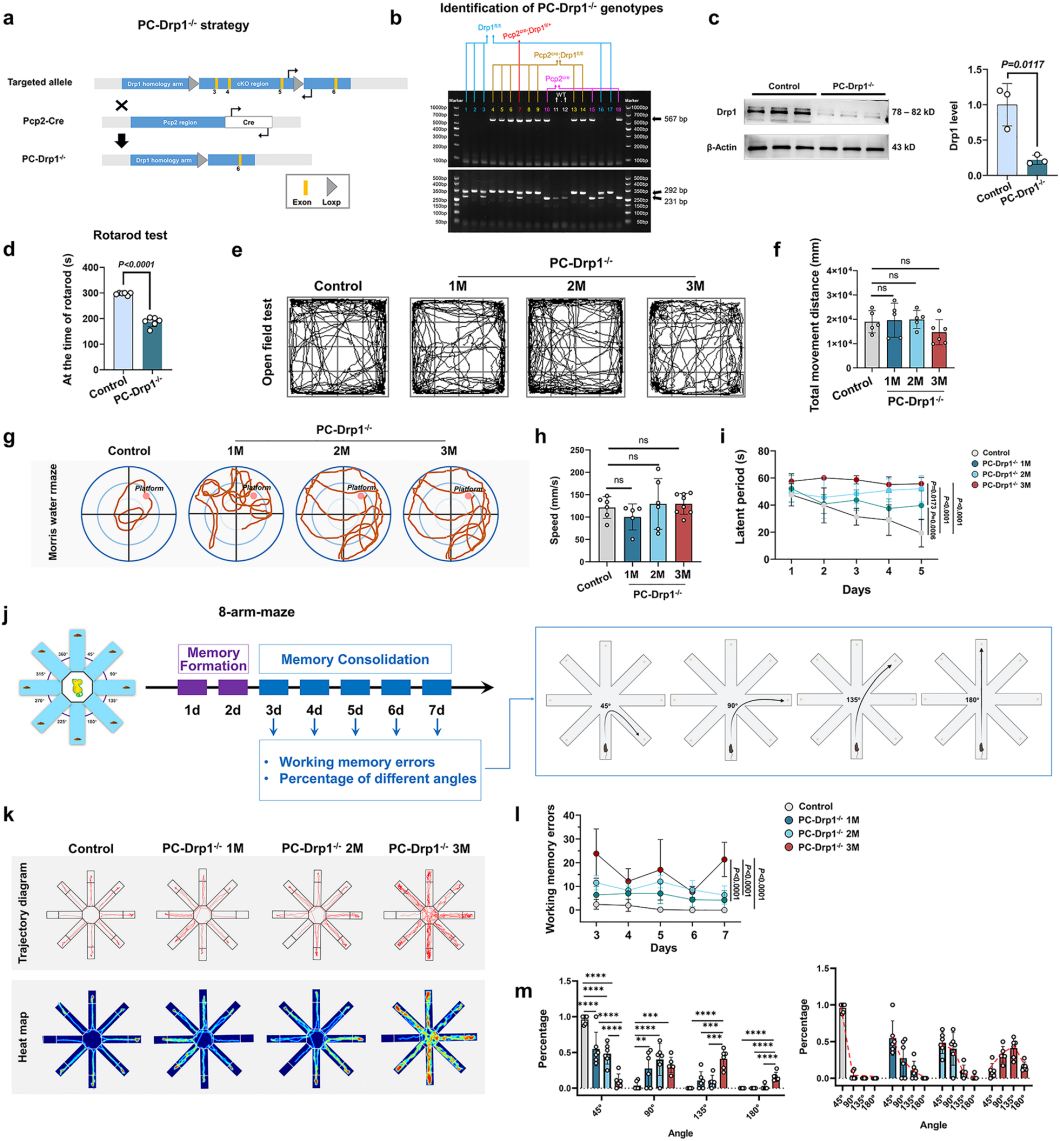
Drp1 deficiency in PCs causes working memory defects in mice. **a** Construction strategy of PC-Drp1^−/−^ mice. **b** Representative diagram of genotyping results. **c** Western blot results and statistical analysis of Drp1 expression level, *n* = 3 mice. **d** Rotarod test results, *n* = 6 mice. **e** Open-field results for one-month-old (1M), two-month-old (2M), and three-month-old (3M) PC-Drp1^−/−^ mice, with 2M Pcp2^Cre^ mice serving as the control group. **f** Quantitative analysis of total movement distance, *n* = 5 or 6 mice. **g** Swimming traces in the water maze. **h** Quantitative analysis of swimming speed, *n* = 5 or 6 mice. **i** Quantitative analysis of the latency, *n* = 5 or 6 mice. **j** Detection strategy for eight-arm maze test in 1M/2M/3M PC-Drp1^−/−^ mice, with Pcp2^Cre^ mice serving as the control group. **k** Traces in the eight-arm maze test. **l** Quantitative analysis of working memory errors, *n* = 5 or 6 mice. **m** Quantitative analysis of percentages of different strategy angles, *n* = 5 or 6 mice. ***P < 0.001, ****P < 0.0001*.* The data are presented as mean ± SD. Unpaired two-tailed *t*-test (**c** and **d**), one-way ANOVA (**f**, **h** and **m**) or two-way ANOVA (**i** and **l**)*.* ns, no significance

**Fig. 3:**
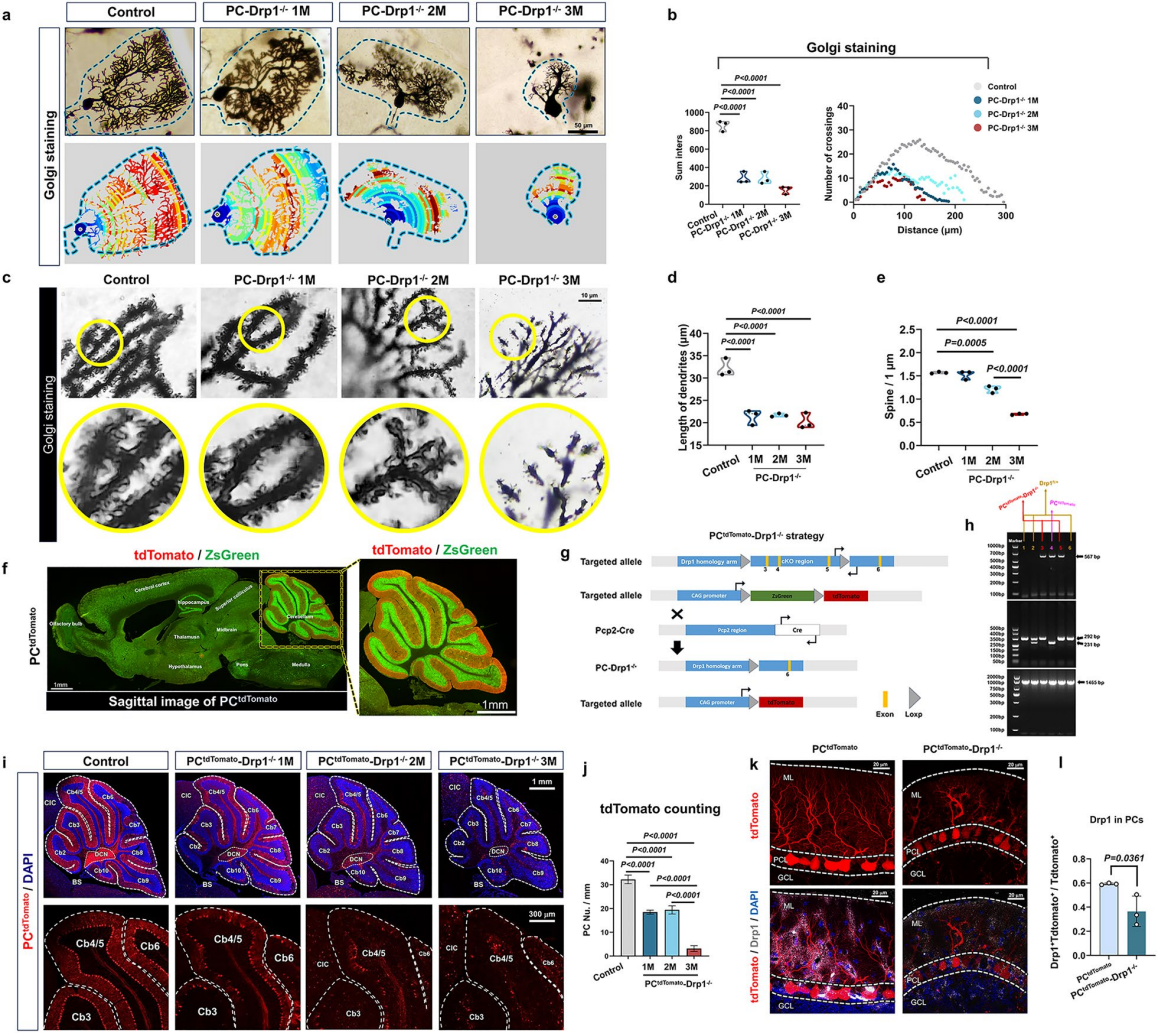
Drp1 deletion reduces PC number and alters dendritic spine morphology. **a** Golgi staining images of PC abundance in 1M/2M/3M PC-Drp1^−/−^ mice, with 2M Pcp2^Cre^ mice serving as the control group. Scale bar, 50 µm.** b** Quantitative analysis results from (**a**), *n* = 3 mice. **c** Golgi staining images of PC dendrite abundance in different groups. Scale bar, 10 µm.** d** Quantitative analysis of distal branch length of PCs, *n* = 3 mice. **e** Quantitative analysis of dendrite spine density of PCs, *n* = 3 mice. **f** Confocal microscopy of a mid-sagittal brain section from a PC^tdTomato^ mouse. Scale bar, 1 mm. **g, h** Construction strategy (**g**) and gene test (**h**) of PC^tdTomato^-Drp1^−/−^ mice. **i** Confocal microscopy of tdTomato-positive cells (PCs) in 1M/2M/3M PC^tdTomato^-Drp1^−/−^ mice, with 2M PC^tdTomato^ mice serving as the control group. Scale bars, 1 mm (upper) and 300 µm (lower). **j** Quantitative results of PC density, *n* = 3 mice. **k** Immunofluorescence for Drp1 in 2M PC^tdTomato^-Drp1^−/−^ mice and PC^tdTomato^ mice. Scale bar, 20 µm.** l** Quantitative analysis of the proportion of cells co-expressing Drp1 and tdTomato relative to the total population of tdTomato-positive cells, *n* = 3 mice. The data are presented as mean ± SD. One-way ANOVA (**b**, **d**, **e** and** j**) or unpaired two-tailed* t*-test (**l**)

**Fig. 4:**
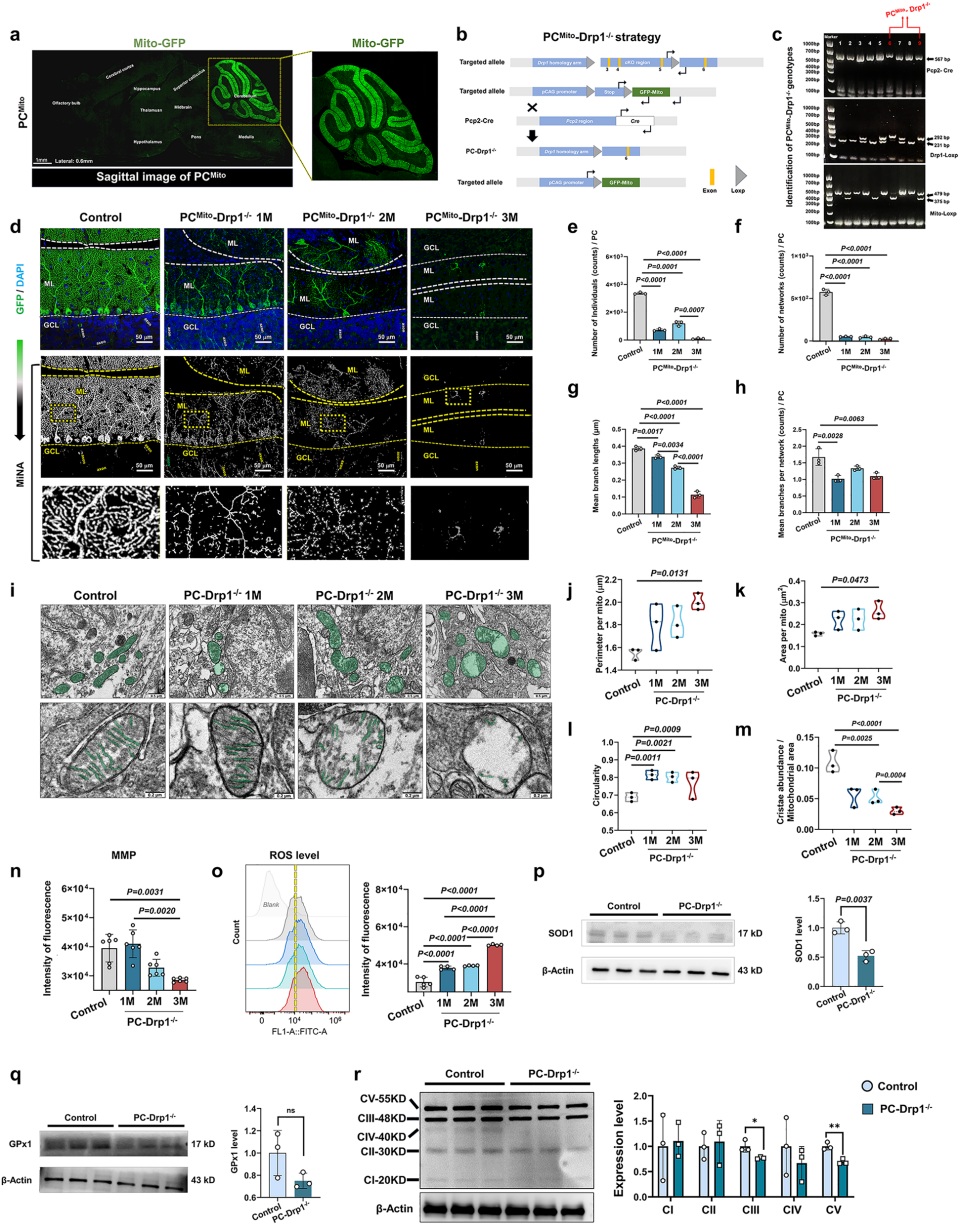
Drp1 defect in PCs severely impairs mitochondrial membrane stability and OXPHOS function. **a** Confocal microscopy of a mid-sagittal brain section from a PC^Mito−GFP^ mouse. Scale bar, 1 mm.** b** Construction strategy of PC^Mito^-Drp1^−/−^ mice. **c** Representative diagram of genotyping results.** d** Confocal microscopy of GFP-positive cells (PCs) in 1M/2M/3M PC^Mito^-Drp1^−/−^ mice, with 2M PC^Mito−GFP^ serving as the control group. Images following MiNA treatment are also provided. Scale bars, 50 µm. **e–h** Quantitative analysis of individual mitochondrial numbers (**e**), network numbers (**f**), network branch length (**g**), and mean branch number of the network (**h**), *n* = 3 mice. **i** Mitochondrial ultrastructure by transmission electron microscopy in all groups. Scale bars, 0.5 µm (upper) and 0.2 µm (lower). **j**–**m** Quantitative analysis of mitochondrial perimeter (**j**), area (**k**), circularity (**l**), and cristae abundance per mitochondrial area (**m**), *n* = 3 mice.** n** Quantitative analysis of mitochondrial membrane potential (MMP) results by JC-1 detection of the purified mitochondria from the cerebellar cortex of 1M/2M/3M PC-Drp1^−/−^ mice, with 2M PC serving as the control group, *n* = 6 mice.** o** ROS results and quantitative analysis by 2’,7’-Dichlorodihydrofluorescein Diacetate (DCFH-DA) detection of the cells isolated from the cerebellar cortex of 1M/2M/3M PC-Drp1^−/−^ mice, with 2M PC serving as the control group, *n* = 4 or 5 mice. **p-r** Western blot analysis for SOD1 (**p**), GPx1 (**q**), and mitochondrial OXPHOS complexes I (NDUFB8), II (SDHB), III (UQCRC2), IV (MTCO1), and V (ATP5A) (**r**) in the cerebellar cortex of 2M PC-Drp1^−/−^ mice and 3M Pcp2^Cre^ control mice, *n* = 3 mice. Mean ± SD, *P < 0.05, **P < 0.01, one-way ANOVA (**e–h** and **j-o**) and unpaired two-tailed *t*-test (**p**–**r**)

Given the established relationship between CoQ10 and Drp1, we employed the Cre-LoxP system to generate three distinct PC-specific Drp1 knockout mouse models. The first model represents a conventional PC-specific Drp1 knockout (PC-Drp1^−/−^ mice) (Fig. 2a). The second model incorporates PC-specific tdTomato reporter expression (PC^tdTomato^-Drp1^−/−^ mice), enabling fluorescent visualization of PCs (Fig. 3g). The third model features mitochondria-targeted GFP expression specifically in PC mitochondria (PC^Mito^-Drp1^−/−^ mice), allowing for direct observation of mitochondrial dynamics in these neurons (Fig. 4b).

Gene analysis (Fig. 2b) indicated that mice numbered 4, 5, 6, 8, 9, 13, and 14 were PC-Drp1^−/−^ mice. Western blot results revealed a 78% decrease in Drp1 protein level (Fig. 2c). Furthermore, immunohistochemical analysis demonstrated that, compared to the control group, PC-Drp1^−/−^ mice exhibited a pronounced reduction in Drp1 protein specifically within PCs, while Drp1 expression levels in NeuN⁺, GFAP⁺, and Iba1⁺ cells remained unchanged (Fig. S2). These results indicated that Drp1 deletion was effectively and selectively targeted to PCs. Observation of PCs in PC^tdTomato^ mice aged 1 to 3 months revealed that the morphology and density of PCs remained stable across these ages (Fig. S3a). An eight-arm maze test conducted on PC^tdTomato^ mice also showed no significant differences in working memory performance among the different age groups (Fig. S3b, c). Together, these findings indicated that both PC architecture and working memory behavioral phenotype remained stable in healthy mice between 1 and 3 months of age. Therefore, in the subsequent experiments, we selected 2-month-old healthy mice as the control group.

The rotarod test results indicated impaired motor coordination in PC-Drp1^−/−^ mice (Fig. 2d). Conversely, the open-field test results confirmed the normal locomotor activity of PC-Drp1^−/−^ mice at 1 month, 2 months, and 3 months of age (Fig. 2e, f). These findings suggested that while PC-Drp1^−/−^ mice exhibited motor incoordination, their ability to walk remained unaffected.

Beginning at one month of age, the Morris water maze test revealed age-dependent progressive impairment of long-term spatial memory in PC-Drp1^−/−^ mice compared to the control mice (Fig. 2g, i). This decline in cognitive function was not attributable to impaired motor function, as evidenced by the normal swimming speeds observed in PC-Drp1^−/−^ mice across all ages (Fig. 2h). Subsequently, working memory was assessed using an eight-arm maze test conducted over seven consecutive days (Fig. 2j, k). The PC-Drp1^−/−^ mice exhibited significant increases in working memory errors at 3 months, but not at 1 or 2 months of age, compared to the control group (Fig. 2l). Moreover, the search strategies of Drp1-deficient mice at all three ages showed a transition from efficient sequential search to non-sequential patterns (Fig. 2m).

### Drp1 deletion reduces PC number and alters the dendritic spine morphology

Golgi staining was performed to compare the complexity of the PC structure between PC-Drp1^−/−^ mice and the control group (Fig. 3a). Sholl’s analysis showed that the complexity of PCs in PC-Drp1^−/−^ mice, as indicated by the number of crossings, was significantly reduced by 70% at both 1 and 2 months and by 90% at 3 months, compared to the control group (Fig. 3b). In-depth analysis of dendritic spine density in the distal dendrites of PCs (Fig. 3c) showed that the lengths of dendrites in PCs were significantly reduced in PC-Drp1^−/−^ mice at all three ages compared to the control group (Fig. 3d). The dendritic spine densities of PCs were significantly reduced in PC-Drp1^−/−^ mice at 2 and 3 months compared to the control group, although no significant difference was observed at 1 month of age (Fig. 3e).

To achieve accurate counting of cerebellar PCs, we generated PC^tdTomato^-Drp1^−/−^ mice by crossing PC-Drp1^−/−^ mice with B6-G/R mice (Fig. 3g). In these transgenic mice, the PCs expressed tdTomato (Fig. 3f). PCR analysis (Fig. 3h) indicated that mice numbered 3 and 5 were the target mice.

Confocal microscopy observation demonstrated that tdTomato expression was exclusively observed in PCs (Fig. 3i). Quantification of tdTomato-positive cells showed a 50% reduction in PC density at both 1 and 2 months, and an approximately 80% reduction at 3 months in PC^tdTomato^-Drp1^−/−^ mice (Fig. 3j). Immunofluorescence staining further revealed the reduction of Drp1 expression in PCs of PC^tdTomato^-Drp1^−/−^ mice (Fig. 3k, l). The levels of PSD95 in the PCs of PC^tdTomato^-Drp1^−/−^ mice were significantly reduced at 2 and 3 months compared to controls (Fig. S4a, b), which were consistent with the changes observed in Golgi-stained dendritic spines. Collectively, our results indicated that PC-specific Drp1 deletion led to age-dependent structural and functional deterioration of PCs, including reduced cell density as well as impaired dendritic arborization and spine density, which was closely associated with the progressive working memory deficits observed in these mice.

### Drp1 defect in PCs severely impairs mitochondrial membrane stability and OXPHOS function

To visualize the mitochondrial morphological changes caused by Drp1 deletion in PCs, we generated PC^Mito^-Drp1^−/−^ mice by crossing PC-Drp1^−/−^ mice with Mito-GFP mice (Fig. 4b). Mice 6 and 9 were identified as the target mice (Fig. 4c). PC^Mito^ mice served as the control. Mitochondria-targeted GFP was exclusively expressed in PCs (Fig. 4a). MiNA revealed significantly reduced mitochondrial interconnectivity and amount in PCs of PC^Mito^-Drp1^−/−^ mice compared to controls (Fig. 4d). Specifically, there were notable decreases in the number of individual mitochondria (Fig. 4e), the number of mitochondrial networks (Fig. 4f), the lengths of mitochondrial branches (Fig. 4g), and the mean branches per mitochondrial network (Fig. 4h). Moreover, the mitochondria in PCs of PC-Drp1^−/−^ mice exhibited significant swelling and severe cristae damage compared to controls (Fig. 4i). Quantitation revealed increased mitochondrial perimeter in 3-month PC-Drp1^−/−^ mice (Fig. 4j); increased mitochondrial area at 3 months (Fig. 4k); increased mitochondrial circularity across all three ages (Fig. 4l); and decreased mitochondrial cristae proportion at both 2 and 3 months (Fig. 4m). MMP in the cerebellum of PC-Drp1^−/−^ mice was significantly reduced at 3 months (Fig. 4n). These results demonstrate that Drp1 deficiency in PCs induces mitochondrial membrane instability.

ROS level in the cerebellum was significantly increased by 1.5 folds at 1 and 2 months, and by 3 folds at 3 months compared to the control (Fig. 4o). Subsequently, we further examined the oxygen consumption rate, glycolytic-related metabolic flux (ECAR), and fatty acid oxidation capacity of the cerebellar tissue from 3-month-old control and PC-Drp1^−/−^ mice. The results showed that compared with the control group, the oxygen consumption rate (Fig. S5a), ECAR (Fig. S5b), and fatty acid oxidation capacity (Fig. S5c) of the cerebellar tissue in Drp1-deficient mice were significantly impaired.

In addition, Western blotting results from cerebellar tissues showed that SOD1 levels were reduced by 50% in 3-month PC-Drp1^−/−^ mice (Fig. 4p), while there was no significant difference in GPx1 level (Fig. 4q). The contents of mitochondrial complex III-UQCRC2 (CIII) and complex V-ATP5A (CV) were significantly reduced, while no significant differences were observed in complex I-NDUFB8, complex II-SDHB and complex IV-MTCO1 (Fig. 4r). These results indicated that Drp1 deficiency in PCs induces severe oxidative stress and OXPHOS dysfunction. Collectively, our results demonstrate that Drp1 deficiency drives PC damage through impairment of mitochondrial membrane stability and OXPHOS function.

### Long-term administration of CoQ10 effectively prevents working memory deficits induced by Drp1 deletion

**Fig. 5:**
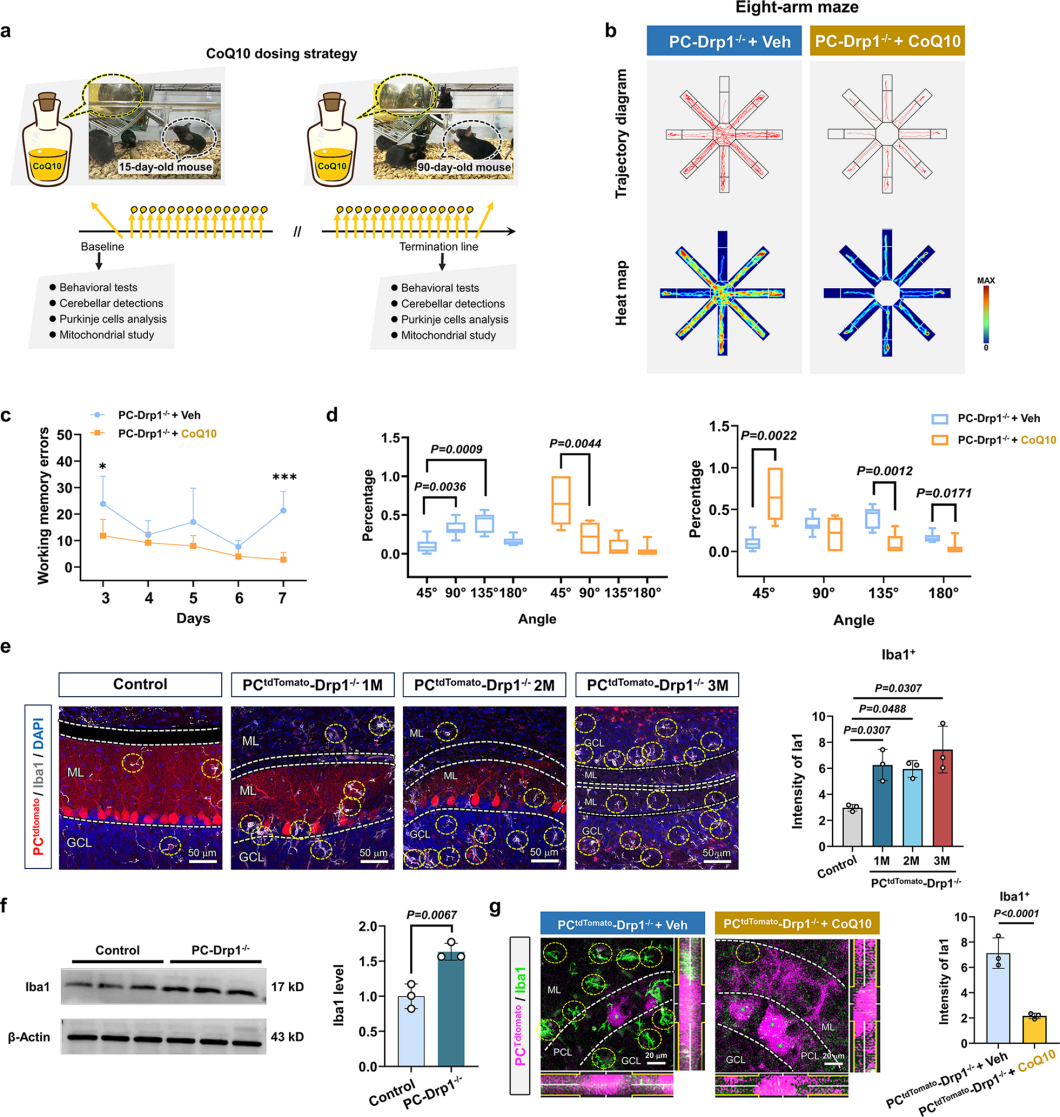
Long-term administration of CoQ10 effectively prevents working memory deficits and cerebellar inflammation. **a** Time line of the experiment. PC-Drp1^−/−^ mice received long-term CoQ10 supplementation or vehicle (PC-Drp1^−/−^ + Veh) in drinking water from postnatal day 15 to day 90. **b** Traces in the eight-arm maze test. **c** Quantitative analysis of working memory errors in the eight-arm maze test, *n* = 5 or 6 mice. **d** Quantitative analysis of food searching strategies, *n* = 5 or 6 mice. **e** Immunofluorescence images and analysis of Iba1 (microglia marker) expression in 1M/2M/3M PC^tdTomato^-Drp1^−/−^ mice, with 2M PC^tdTomato^ mice serving as the control group. Scale bars, 50 µm, *n* = 3 mice.** f** Western blot analysis for Iba1 in 3M PC-Drp1^−/−^ mice and 3M Pcp2^Cre^ control mice, *n* = 3 mice. **g** Immunofluorescence images and quantitative analysis of Iba1 (green) expression in PCs (red) of PC^tdTomato^-Drp1^−/−^ + CoQ10 group and PC^tdTomato^-Drp1^−/−^ + Veh group. Scale bars, 20 µm, *n* = 3 mice. Mean ± SD, two-way ANOVA (**c**), one-way ANOVA (**d** and **e**) and unpaired two-tailed* t*-test (**f** and **g**)

To verify the therapeutic effect of CoQ10 in Drp1-deficient diseases, PC-Drp1^−/−^ mice, PC^tdTomato^-Drp1^−/−^ mice, and PC^Mito^-Drp1^−/−^ mice received long-term administration of CoQ10 continuously via drinking water supplementation from age 15 days till 90 days (Fig. 5a). CoQ10 was dissolved in a 3% DMSO solution at a concentration of 500 µM. The mice in the control group were provided with drinking water containing 3% DMSO (Vehicle, Veh). To rule out the interference of DMSO on the drinking behavior or basal metabolism of mice, we fed PC-Drp1^−/−^ mice with pure water, 3% DMSO, and 500 µM CoQ10 solution respectively and conducted metabolic cage experiments. The results showed that compared with the pure water group, the 3% DMSO group and the CoQ10 group showed no significant differences in the water intake (Fig. S6a, b), oxygen consumption (VO₂) (Fig. S6c, d), carbon dioxide production (VCO₂) (Fig. S6e, f), RER (Fig. S6g, h), and EE (Fig. S6i, j). This result supported that 3% DMSO had no significant effect on the basal metabolism and drinking behavior.

To address the concern that there might be differences in individual CoQ10 (500 µM) intake under group-living conditions, we separately measured the CoQ10 contents in the serum and cerebellum tissues of 5 mice housed in the same cage. The results showed that the CoQ10 levels in the serum (Fig. S7a) and cerebellar tissue (Fig. S7b) of these 5 mice in the same cage were highly consistent, with no significant differences. Therefore, the subsequent experiments were conducted by supplementing CoQ10 through drinking water. To determine the concentration of CoQ10 in the experimental mice, we fed PC-Drp1^−/−^ mice with 3% DMSO or 500 µM CoQ10 for one month. Then, we measured the concentrations of CoQ10 in the serum and cerebellar tissue of the mice. The results showed that compared with the vehicle group, the average levels of CoQ10 in the serum (Fig. S8a) and cerebellar tissue (Fig. S8b) were significantly increased in the CoQ10 group.

Behavioral analyses demonstrated that CoQ10 treatment reduced working memory errors (Fig. 5b, c). Additionally, long-term supplementation with CoQ10 enhanced the highly efficient exploratory strategy based on sequential search (Fig. 5b, d). Immunofluorescence staining revealed increased microglial activation in Drp1-deficient mice at 1, 2, and 3 months compared to the control group (Fig. 5e). The Western blotting analysis further confirmed the increased level of microglial marker Iba1 in cerebellar tissue (Fig. 5f). Following the CoQ10 intervention, the number of activated microglia was reduced by 60% (Fig. 5g).

Moreover, to investigate whether CoQ10 is harmful to healthy mice, WT mice received 1-month CoQ10 water supplementation (concentrations including 250 μM, 500 μM, and 1000 μM). We did not observe any significant difference in PC morphology (Fig. S9a), mitochondrial quantity (Fig. S9a, b), working memory (Fig. S9c, d), ROS level (Fig. S9e, f), ATP content (Fig. S9g), or MMP level (Fig. S9h) compared to the control groups (water or DMSO). This indicated that under our experimental conditions, long-term supplementation of CoQ10 did not cause any significant damage to healthy mice.

These findings indicated that long-term CoQ10 supplementation prevents working memory deficits in mice with PC-specific conditional deletion of Drp1.

### CoQ10 treatment effectively attenuates PC dysfunction induced by Drp1 deficiency

**Fig. 6:**
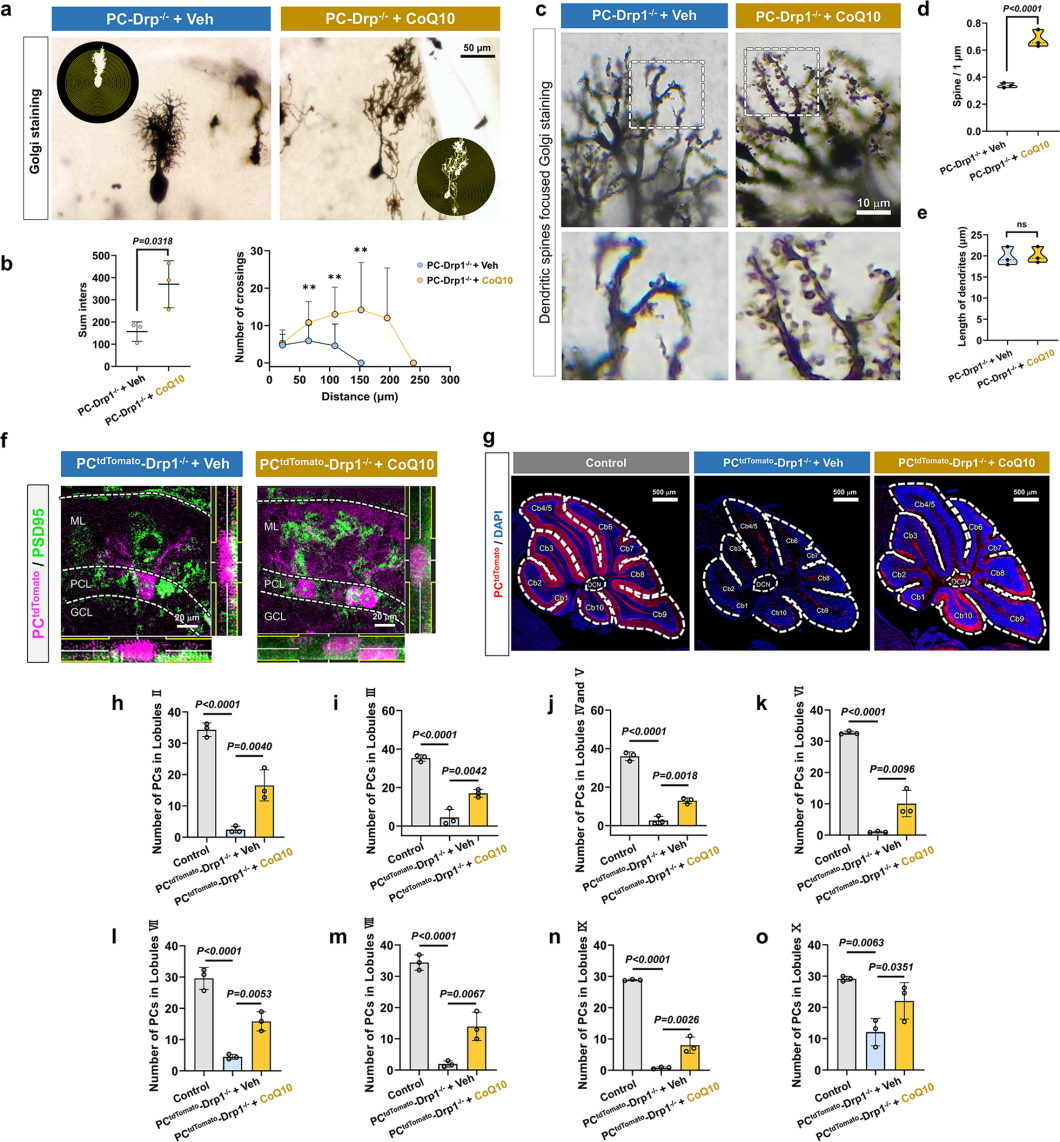
CoQ10 treatment prevents impairment of PC structure induced by Drp1 deletion. **a** Golgi staining images of PCs in PC-Drp1^–/–^ with long-term CoQ10 supplementation or vehicle. Scale bar, 50 µm.** b** Quantitative analysis of the complexity of PCs, *n* = 3 mice. **c** Golgi staining images of PC dendrite abundance in PC-Drp1^–/–^ + CoQ10 group and PC-Drp1^–/–^ + Veh groups. Scale bar, 10 µm. **d, e** Quantitative analysis of the density of PC dendritic spines (**d**) and dendritic branch length (**e**), *n* = 3 mice. **f** Immunofluorescence images of PSD95 expression in PC^tdTomato^-Drp1^–/–^ + CoQ10 and PC^tdTomato^-Drp1^–/–^ + Veh groups. Scale bars, 20 µm.** g** Confocal microscopy images of tdTomato-positive cells (PCs) in PC^tdTomato^-Drp1^–/–^ + CoQ10 group and PC^tdTomato^-Drp1^–/–^ + Veh group. Scale bars, 500 µm. **h–o** Quantitative results of PC density in lobules II–X, *n* = 3 mice. Mean ± SD, unpaired two-tailed *t*-test (**b**, **d** and** e**) and one-way ANOVA (**h**–**o**). ns, no significance

Significant increases in both the number and the complexity of PC dendritic branches were observed following the CoQ10 intervention (Fig. 6a, b). Further analysis of PC dendritic spines revealed that CoQ10 increased dendritic spine density (Fig. 6c, d); however, no statistical difference was found in dendritic branch length (Fig. 6c, e). Additionally, both PSD95 expression (Fig. 6f, and Fig. S4c, d) and PC density (Fig. 6g) were improved markedly following CoQ10 treatment. To further confirm whether CoQ10 completely attenuated the damage to the PCs in the cerebellum of PC^tdTomato^-Drp1^–/–^ mice, we separately counted the numbers of PCs in cerebellar lobules II-X. The results showed that although the total number of PCs in the CoQ10-treated group remained lower than that in the healthy control, CoQ10 administration significantly slowed the rate of PC loss across all cerebellar lobules in Drp1-deficient mice (Fig. 6h-o).

We further recorded the electrical activity of PCs using the membrane clamp technique. No significant differences were observed in access resistance (*R*a) (Fig. S10a, b) or membrane resistance (*R*m) (Fig. S10a, c), indicating that Drp1 knockout did not disrupt the integrity of the PC membrane. However, the PC-Drp1^−/−^ mice showed significant decreases in the membrane time constant (tau) and membrane capacitance (*C*m), indicative of neuronal atrophy and dendritic spine loss, which were reversed by CoQ10 intervention (Fig. S10d, e). These findings were consistent with the Golgi staining results. Additionally, in the voltage-clamp mode, we observed a significantly increased current response to hyperpolarization activation in the PC-Drp1^−/−^ mice. This abnormality was reversed in the CoQ10-treated group (Fig. S10f, g). The observed PC dysfunction may be attributed to the aberrant activation of hyperpolarization-activated cyclic nucleotide-gated (HCN) channels. To directly test whether HCN channels mediate the therapeutic effects of CoQ10 in PC-Drp1^−/−^ mice, we locally administered the selective HCN channel inhibitor ZD7288 via an implanted cannula (Fig. S10h). Subsequent eight-arm maze testing revealed that, at the dosage and paradigm used, ZD7288 did not significantly alter working memory performance in these mice (Fig. S10i). Under the current experimental conditions, HCN channel blockade did not demonstrate behavioral benefits, suggesting that HCN channels are unlikely the primary determinant for the CoQ10-mediated phenotypic improvement.

### Thermal proteome profiling identified key target proteins of CoQ10 in Drp1-deficient mice

**Fig. 7:**
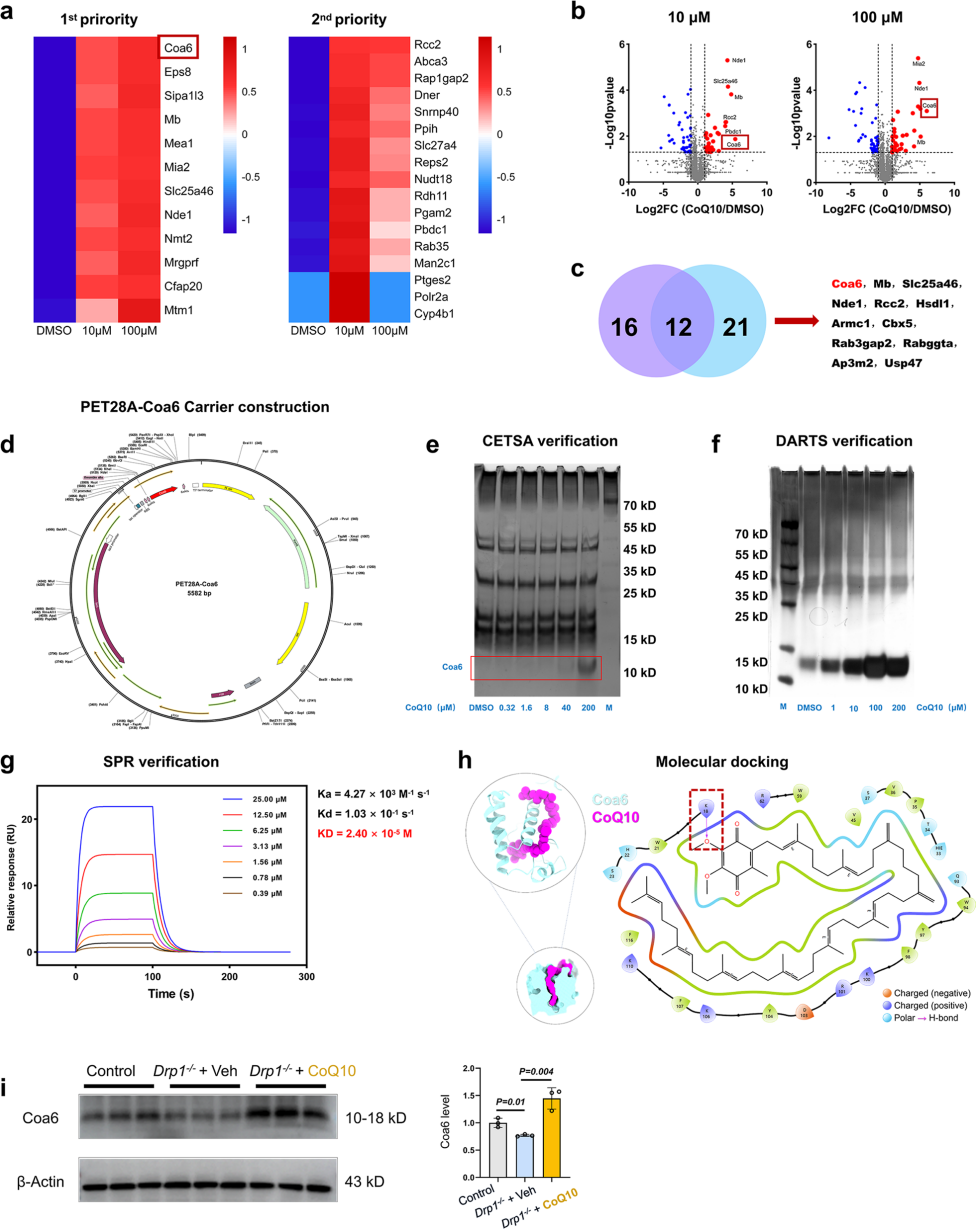
CoQ10 ameliorates memory impairment in DRP1-deficient mice by binding to Coa6. **a** Proteomic identification and analysis by Orbitrap HF-X LC–MS/MS high-resolution mass spectrometry. HFX was used for mass spectrometry signal acquisition (with a 70-min time gradient for each sample), *n* = 3 mice. **b** Differential gene analysis between the 10 μM and 100 μM CoQ10 groups and their respective control groups**. c** The intersection of the two groups of heat-resistant proteins was analyzed. **d** PET28A-Coa6 was constructed into the prokaryotic expression vector PET28A by homologous recombination method. **e**–**g** Qualitative analysis of Coa6 content by CETSA (**e**), DARTS (**f**), and surface plasmon resonance (**g**), *n* = 3 mice. **h** The structure of the Coa6-CoQ10 complex was predicted using ProtENIX. In this three-dimensional schematic diagram, orange-marked regions denote negatively charged binding sites, bluish violet regions denote positively charged binding sites, and purple arrows indicate directional hydrogen bonding with the arrow tail representing the hydrogen bond donor and the arrowhead pointing to the hydrogen bond acceptor. These three types of molecular interactions collectively constituted key binding elements at the CoQ10-Coa6 interaction interface. The red dotted box shows that the methoxy group of CoQ10 forms a hydrogen bond with the lysine residue at position 18 (K18) of Coa6, while its extended hydrophobic tail engages in hydrophobic interactions with residues including Tryptophan at position 59 (W59), Valine at position 45 (V45), and Proline at position 35 (P35), the 36th Valine (V36), the 94th Tryptophan (W94), the 97th Tyrosine (Y97), the 98th Phenylalanine (F98), the 104th Tyrosine (Y104), the 107th Phenylalanine Residues (F107) of Coa6. **i** Western blot analysis for Coa6 in the cerebellar cortex of Control, PC-Drp1^−/−^ + CoQ10 mice and PC-Drp1^−/−^ + Veh mice, *n* = 3 mice. Mean ± SD, one-way ANOVA (**i**)

To identify the molecular target of CoQ10 in Drp1-induced mitochondrial disease, we conducted a small molecule drug target fishing experiment. First, we conducted thermal proteome profiling analysis and divided the data into two priorities. We identified 12 candidate targets in the first priority, which showed significantly different levels between the 10 μM CoQ10 and the DMSO group and the levels increased with increasing CoQ10 concentrations. Seventeen proteins were identified in the second priority (Fig. 7a), whose levels showed a presence-versus-absence difference compared to the DMSO group, but did not increase with increasing CoQ10 concentrations.

Second, through analysis of the differences between the low-concentration and the control group, 28 significant heat-resistant proteins were screened out. Analysis of the differences between the high-concentration and the control group screened out 33 significant heat-resistant proteins (Fig. 7b). Taking the overlap of the two groups of heat-resistant proteins, 12 common proteins were found (Fig. 7c), namely Coa6, Mb (Myoglobin), Slc25a46 (Solute carrier family 25 member 46), Nde1 (NudE neurodevelopment protein 1), Rcc2 (Regulator of chromosome condensation 2), Hsdl1 (Hydroxysteroid dehydrogenase like 1), Armc1 (Armadillo repeat-containing protein 1), Cbx5 (Chromobox homolog 5), Rab3gap2 (RAB3 GTPase activating protein subunit 2), Rabggta (Rab geranylgeranyl transferase), Ap3m2 (Adaptor-related protein complex 3, mu 2 subunit), and Usp47 (ubiquitin specific peptidase 47). In the first-priority data, the Coa6 protein was also selected as the candidate validation target protein. Therefore, we generated the PET28A-Coa6 protein vector to produce the protein (Fig. 7d).

### CoQ10 rescues memory impairment in Drp1-deficient mice by binding to Coa6

To validate Coa6 as a target protein of CoQ10, we assessed the thermal stability and protease resistance of Coa6 using CETSA and DARTS assays, respectively. The CETSA experiment showed that the Coa6 protein had a significantly increased thermal stability, that was, with increasing CoQ10 concentrations, the Coa6 protein showed increased retention (Fig. 7e). The DARTS data further revealed a dose-dependent protection of Coa6 against proteolysis, with increasing CoQ10 concentrations correlating with enhanced protein stability (Fig. 7f). To quantitatively analyze the binding affinity between CoQ10 and Coa6, we further conducted surface plasmon resonance experiments. The results revealed specific binding between CoQ10 and CM5-immobilized Coa6, yielding a dissociation constant (*K*_D_) of 2.40 × 10^–5^ M (Fig. 7g). These results demonstrated high-affinity binding between Coa6 and CoQ10.

We predicted the structure of the Coa6-CoQ10 complex using ProtENIX. Molecular docking prediction indicated that CoQ10 is embedded in the protein-binding pocket of Coa6, forming a strong hydrophobic complementarity. The methoxy group of CoQ10 forms a hydrogen bond with the 18th Lysine residue (K18) of Coa6, and its extended hydrophobic tail establishes hydrophobic interactions with residues including Tryptophan at position 59 (W59), Valine at position 45 (V45), and Proline at position 35 (P35), the 36th Valine (V36), the 94th Tryptophan (W94), the 97th Tyrosine (Y97), the 98th Phenylalanine (F98), the 104th Tyrosine (Y104), and the 107th Phenylalanine Residue (F107). These findings provide strong evidence for the molecular interaction model between Coa6 and CoQ10 (Fig. 7h).

Furthermore, Western blotting results showed that the expression of Coa6 in the cerebellum of PC-Drp1^−/−^ mice markedly decreased (Fig. 7i). Immunofluorescence staining revealed significant reductions of Coa6 expression in PCs of PC-Drp1^−/−^ mice (Fig. S11a, b). Meanwhile, qPCR analysis indicated that the relative mRNA expression of Coa6 did not significantly change under the condition of Drp1 deficiency (Fig. S11c), suggesting that the downregulation of Coa6 was not caused by transcriptional regulation. To further assess the impact of Drp1 deficiency on the stability of the Coa6 protein, we conducted a cellular thermal shift assay in the cerebellar tissues (tissue-CETSA) of Drp1-deficient and control mice. The results showed that under the condition of Drp1 deficiency, the Coa6 protein became insoluble or degraded earlier under gradually increased heat, suggesting a significant decrease in the thermal stability of Coa6 (Fig. S11d). These results collectively support the conclusion that Drp1 deficiency mainly reduces the stability of Coa6 protein rather than the transcriptional level of Coa6, leading to decreased protein level.

Moreover, Western blotting showed that the Coa6 protein level significantly increased after CoQ10 intervention (Fig. 7i). The Coa6 thermal stability in the cerebellum tissues of CoQ10-fed PC-Drp1^−/−^ mice was enhanced (Fig. S8c). According to prior research [34, 35], reduced Coa6 expression disrupts the complex IV assembly, consequently diminishing complex IV activity and OXPHOS function. CoQ10 stabilized Coa6, which may facilitate the assembly of mitochondrial Complex IV within the mitochondrial inner membrane. Further studies are needed to determine whether CoQ10 binding to Coa6 enhances mitochondrial OXPHOS function.

### CoQ10 improves mitochondrial membrane stability and OXPHOS complexes III-V activity under condition of Drp1 deficiency

**Fig. 8:**
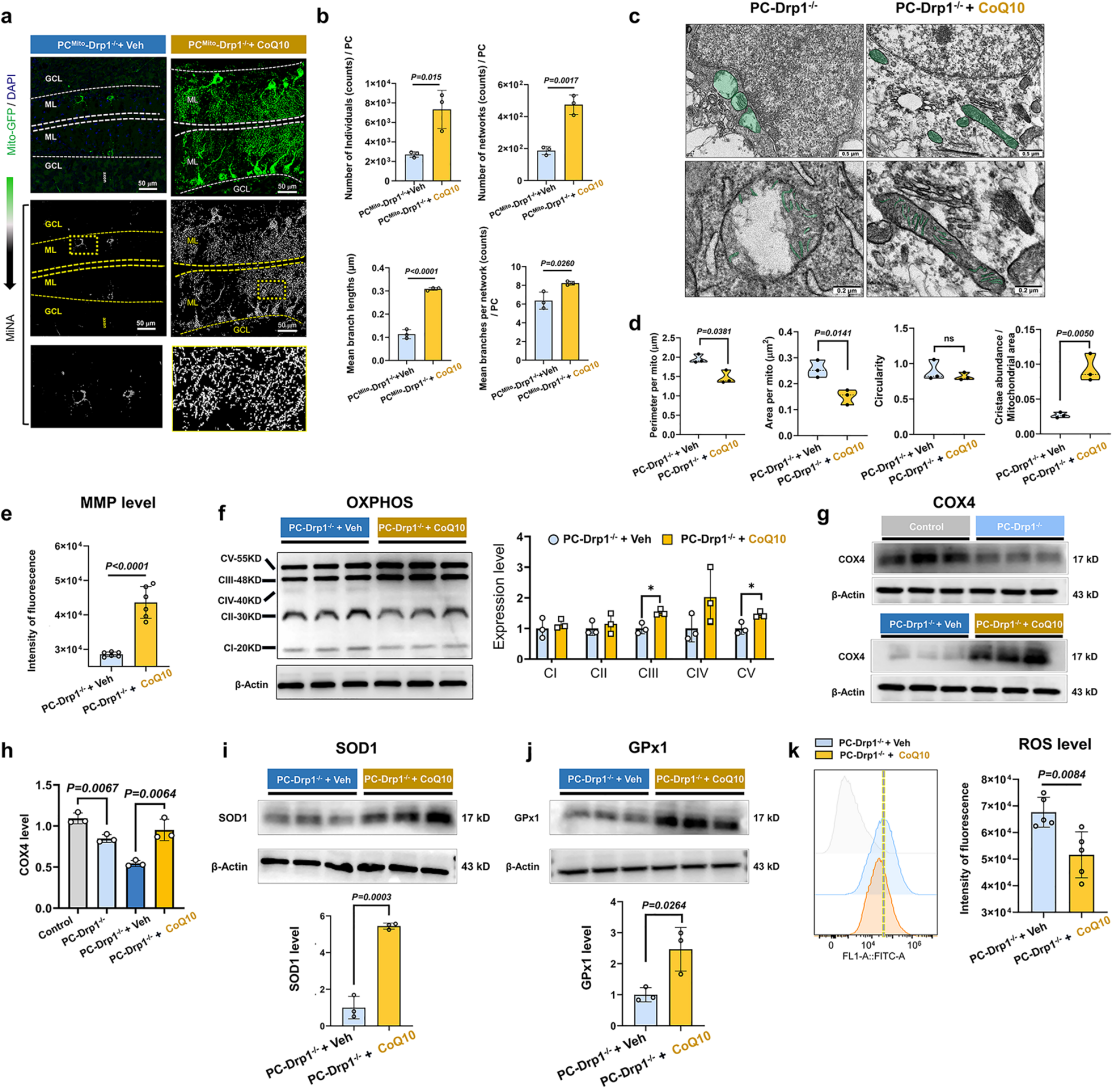
CoQ10 ameliorates Drp1 deficiency-induced mitochondrial membrane instability and ETC dysfunction. **a** Confocal microscopy images of GFP-positive cells (PCs) in PC^Mito^-Drp1^−/−^ mice with long-term CoQ10 or vehicle treatment. Images following MiNA treatment are also provided. Scale bars, 50 µm.** b** Quantitative analysis of the individual mitochondrial number, mitochondrial network, mitochondrial network branch length, and mean branch quantity of the mitochondrial network in PC^Mito^-Drp1^−/−^ + CoQ10 group and PC^Mito^-Drp1^−/−^ + Veh group, *n* = 3 mice. **c** Mitochondrial ultrastructure by transmission electron microscopy. Scale bars, 0.5 µm (upper) and 0.2 µm (lower). **d** Quantitative analysis of mitochondrial perimeter, area, circularity, and cristae abundance, *n* = 3 mice. **e** Quantitative analysis of mitochondrial membrane potential (MMP) results by JC-1 detection of the purified mitochondria from the cerebellar cortex of PC-Drp1^−/−^ + CoQ10 mice and PC-Drp1^−/−^ + Veh mice, *n* = 6 mice. **f** Western blot analysis for mitochondrial OXPHOS complexes I (NDUFB8), II (SDHB), III (UQCRC2), IV (MTCO1), and V (ATP5A), in the cerebellar cortex of PC-Drp1^−/−^ + CoQ10 mice and PC-Drp1^−/−^ + Veh mice, *n* = 3 mice. *P < 0.05. **g-j** Western blot analysis for COX4 (**g, h**), SOD1 (**i**) and GPx1 (**j**) in PC-Drp1^−/−^ + CoQ10 mice and PC-Drp1^−/−^ + Veh mice, *n* = 3 mice. **k** ROS results and quantitative analysis by DCFH-DA detection of the cells isolated from the cerebellar cortex of PC-Drp1^−/−^ + CoQ10 mice and PC-Drp1^−/−^ + Veh mice, *n* = 5 mice. Mean ± SD, unpaired two-tailed *t*-test (**b**, **d**–**f** and **h–k**)

Given that Coa6 is an important subunit of mitochondrial complex IV, we further examined the effects of CoQ10 on mitochondrial morphology and respiratory function in PC-Drp1^−/−^ mice. Analysis with MiNA revealed that CoQ10 intervention increased the number of individual mitochondria, mitochondrial networks, branching complexity, and branch length in PCs (Fig. 8a, b). Electron microscopy analysis of cerebellar tissue showed that CoQ10 decreased the mitochondrial perimeter and area while increasing the respiratory cristae occupancy (Fig. 8c, d). Additionally, MMP was elevated in cerebellar tissue following CoQ10 supplementation (Fig. 8e). These results demonstrated that CoQ10 corrected the Drp1 deficiency-induced mitochondrial membrane instability.

Furthermore, CoQ10 intervention selectively increased the protein levels of respiratory chain complexes III (UQCRC2) and V (ATP5A), with no significant changes in complexes I (NDUFB8), II (SDHB), and IV (MTCO1) (Fig. 8f). We further quantified COX4 expression and found that CoQ10 significantly rescued the Drp1 deficiency-induced reduction in COX4 levels (Fig. 8g, h). CoQ10 supplementation significantly increased levels of the antioxidant enzymes SOD1 (Fig. 8i) and GPx1 (Fig. 8j) in cerebellar tissue. Quantitative ROS assays confirmed reduced ROS in this region in PC-Drp1^−/−^ mice (Fig. 8k). Metabolic analysis of cerebellar tissue revealed significant increases in the oxygen consumption rate (Fig. S5d), ECAR (Fig. S5e), and fatty acid oxidation activity (Fig. S5f) in PC-Drp1^−/−^ mice receiving CoQ10 treatment compared with vehicle treatment. These results demonstrated that CoQ10 corrected the Drp1 deficiency-induced oxidative stress and OXPHOS dysfunction.

In conclusion, CoQ10 restored mitochondrial membrane stability and OXPHOS function, specifically complexes III, IV, and V, at least in part by binding to Coa6 and elevating its expression. Additionally, the improvement in complex IV function was dependent on the Coa6 assembly factor rather than the structural MTCO1 subunit.

### Coa6 upregulation in PCs rescues the Drp1 deficiency-induced working memory impairment and mitochondrial dysfunction

**Fig. 9:**
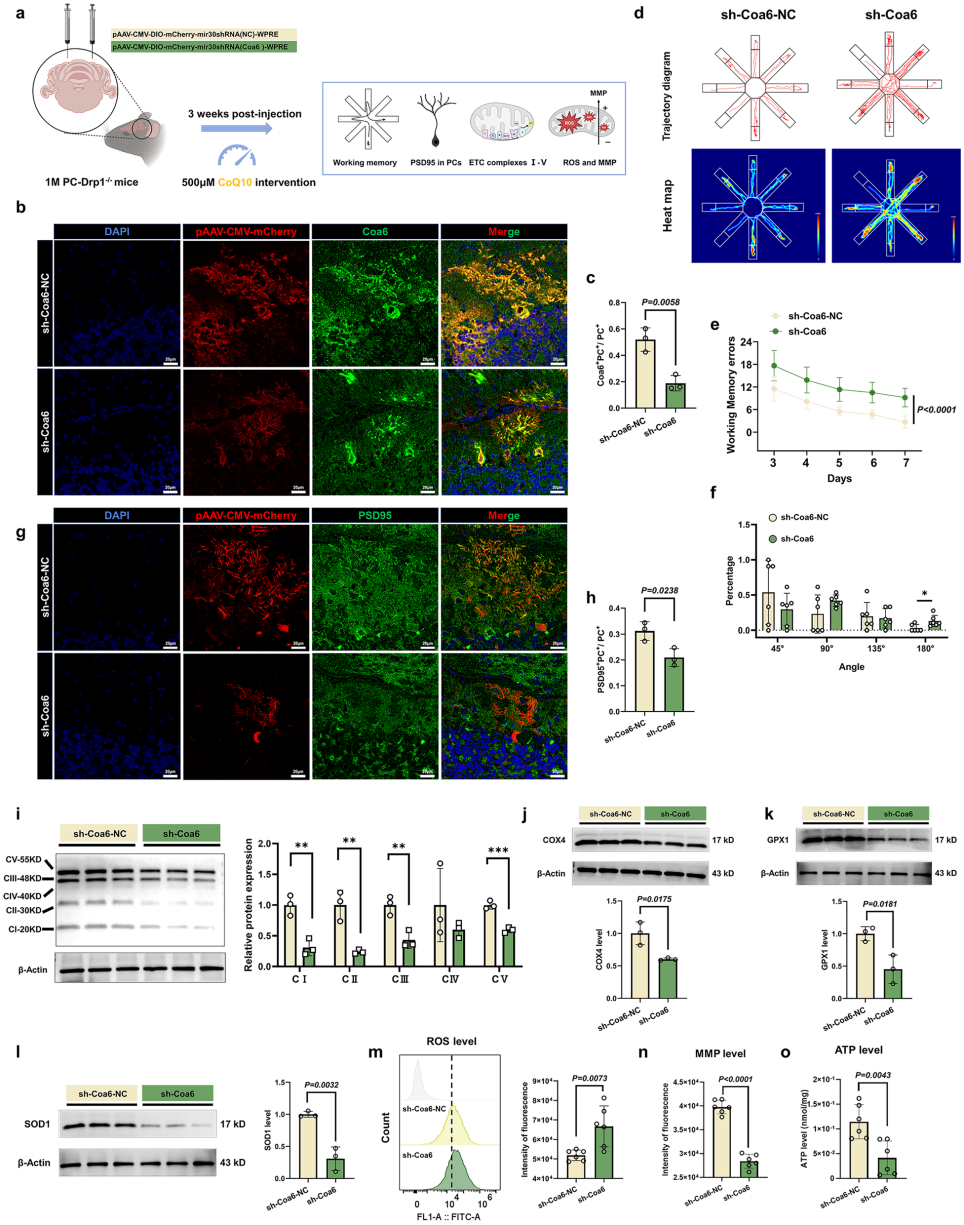
Down-regulation of Coa6 abolishes the therapeutic efficacy of CoQ10 in Drp1-deficient mice. **a** Schematic of the experimental design for Drp1-deficient mice after Coa6 down-regulation. **b**, **c** Multiple immunohistochemical images (**b**) and quantification (**c**) of Coa6 expression in the PCs. Scale bars, 20 µm. *n* = 3 mice. **d** Traces in the eight-arm maze test. **e** Quantitative analysis of working memory errors, *n* = 6 mice. **f** Quantitative analysis of percentages of different strategy angles, *n* = 6 mice. *P < 0.05*.* **g, h** Multiple immunohistochemical images (**g**) and quantification (**h**) of PSD95 expression in the PCs. Scale bars, 20 µm. *n* = 3 mice. **i-l** Western Blot analysis of the expression levels of mitochondrial OXPHOS complexes (**i**), COX4 (**j**), GPX1 (**k**), and SOD1 (**l**) in the cerebellum, *n* = 3 mice. **P < 0.01, ***P < 0.001*.* **m** Quantitative analysis of ROS results using DCFH-DA detection in the cerebellum, *n* = 6 mice. **n** Quantitative analysis of mitochondrial membrane potential (MMP) results by JC-1 detection in the cerebellum, *n* = 6 mice. **o** Quantitative analysis of ATP results in the cerebellum, *n* = 6 mice. Mean ± SD, unpaired two-tailed *t*-test (**c**, **f**, **h–o**) and two-way ANOVA (**e**)

To delineate the functional role of Coa6 in the context of Drp1 deficiency, we performed Coa6 knockdown and overexpression experiments in PC-Drp1^−/−^ mice. In the Coa6 knockdown study, one-month-old PC-Drp1^−/−^ mice were randomly assigned to receive either control (sh-Coa6-NC) or Coa6-targeting (sh-Coa6) viruses, followed by CoQ10 supplementation in water for three weeks (Fig. 9a). Multiple immunohistochemical staining confirmed efficient Coa6 knockdown in PCs (Fig. 9b, c). Behavioral assessment in the eight-arm maze revealed that Coa6 knockdown significantly abrogated the protective effect of CoQ10, as evidenced by increased working memory errors (Fig. 9d–f). Furthermore, Coa6-deficient PCs exhibited a marked reduction in dendritic spine density, as indicated by PSD95 immunostaining (Fig. 9g, h). At the mitochondrial level, Coa6 knockdown led to a pronounced decrease in the protein levels of OXPHOS complexes I–V (Fig. 9i, j) and antioxidant enzymes GPX1 and SOD1 (Fig. 9k, l). This was accompanied by elevated ROS production (Fig. 9m), loss of MMP (Fig. 9n), and a reduction in ATP content (Fig. 9o).

**Fig. 10:**
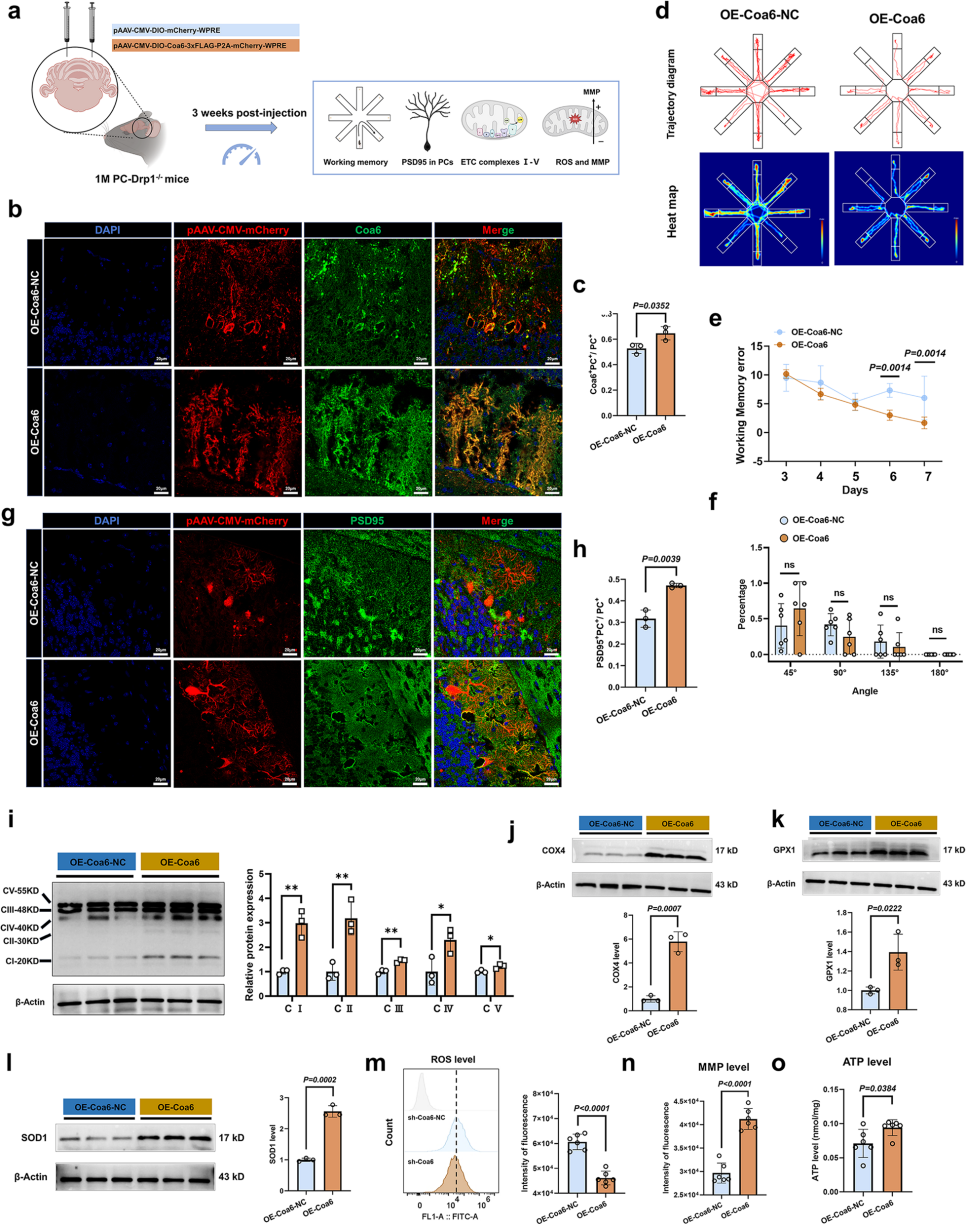
Coa6 up-regulation rescues the Drp1 deficiency-induced working memory impairment and mitochondrial dysfunction. **a** Schematic of the experimental design for Drp1-deficient mice after Coa6 up-regulation. **b, c** Multiple immunohistochemical images (**b**) and quantification (**c**) of Coa6 expression in PCs. Scale bars, 20 µm. *n* = 3 mice. **d** Traces in the eight-arm maze test. **e** Quantitative analysis of working memory errors, *n* = 6 mice. **f** Percentages of different strategy angles, *n* = 6 mice. **g, h** Multiple immunohistochemical images (**g**) and quantification (**h**) of PSD95 in PCs. Scale bars, 20 µm. *n* = 3 mice. **i-l** Western blot analysis for mitochondrial OXPHOS complexes (**i**), COX4 (**j**), GPX1 (**k**), and SOD1 (**l**) in the cerebellum, *n* = 3 mice. *P < 0.05, **P < 0.01. **m** Quantitative analysis of ROS results by DCFH-DA detection in the cerebellum, *n* = 6 mice. **n** Quantitative analysis of mitochondrial membrane potential (MMP) by JC-1 detection in the cerebellum, *n* = 6 mice. **o** Quantitative analysis of ATP results in the cerebellum, *n* = 6 mice. Mean ± SD, unpaired two-tailed *t*-test (**c, f, h**–**o**) and two-way ANOVA (**e**)

Conversely, in the Coa6 overexpression study, one-month-old PC-Drp1^−/−^ mice received either control (OE-Coa6-NC) or Coa6-overexpression (OE-Coa6) viruses and were maintained on regular water (without CoQ10) for three weeks (Fig. 10a). Successful Coa6 overexpression in PCs was verified by multiple immunohistochemical staining (Fig. 10b, c). Importantly, overexpression of Coa6 alone partially improved the pathological phenotype of PC-Drp1^−/−^ mice. This improvement included fewer working memory errors (Fig. 10d–f), restoration of PC dendritic spine density (Fig. 10g, h), increased protein levels of mitochondrial complexes I–V (Fig. 10i, j), and improved mitochondrial function, reflected as higher MMP and ATP levels (Fig. 10n, o), alongside attenuated oxidative stress (Fig. 10k–m).

Critically, to determine if the effects of Coa6 were secondary to alterations of residual Drp1 levels, we assessed Drp1 protein levels following Coa6 modulation. Neither Coa6 knockdown nor overexpression altered Drp1 levels in PCs of PC-Drp1^−/−^ mice (Fig. S11e, h).

Collectively, these findings demonstrated that Coa6 plays an active regulatory role in the associated pathology. Moreover, Coa6 mediates the mitochondrial protective effects of CoQ10, positioning it as a critical molecular effector downstream of Drp1 deficiency.

## Discussion

### CoQ10 attenuates the respiratory chain defects and working memory decline caused by Drp1 loss in PCs by binding to Coa6

**Fig. 11:**
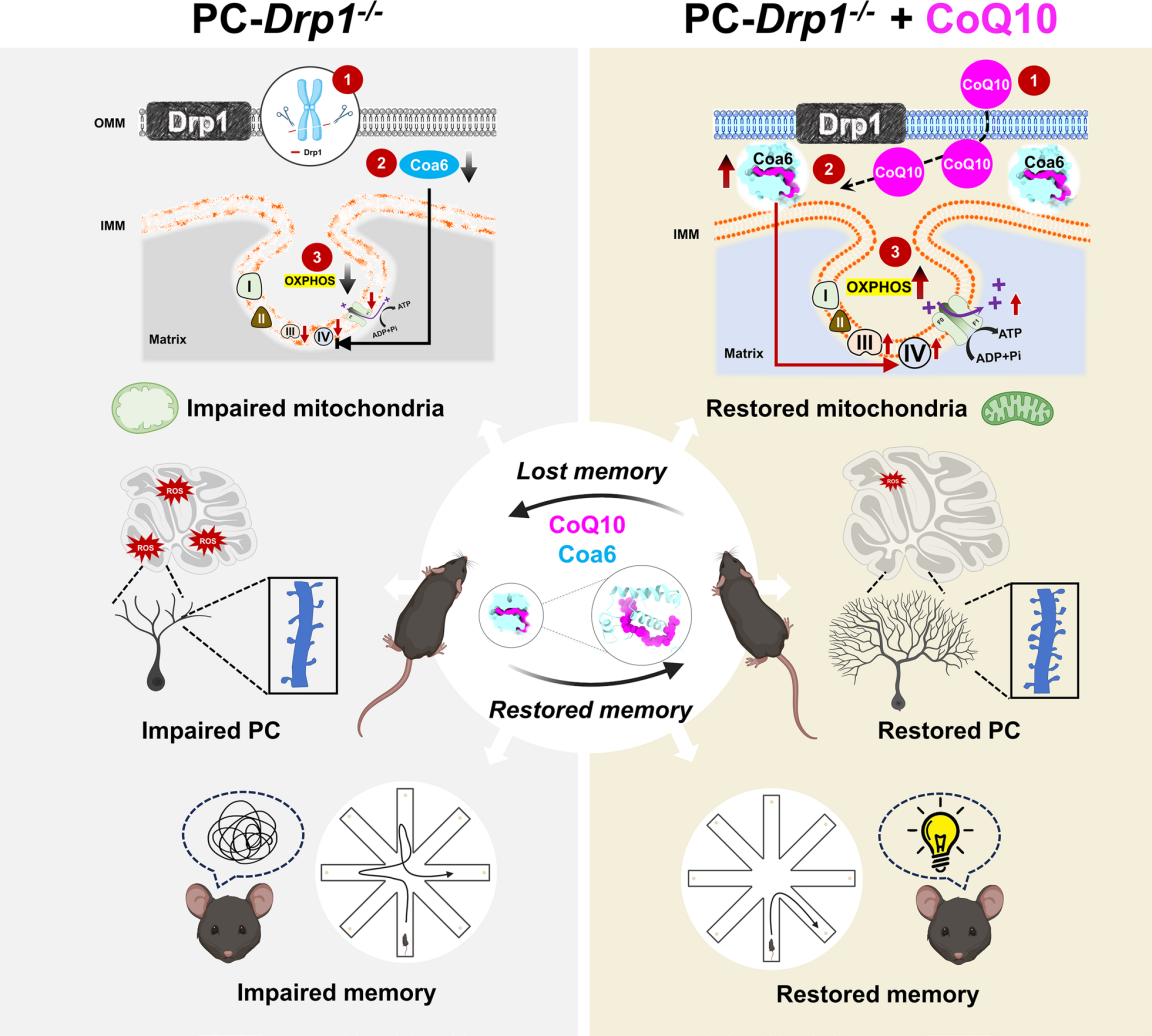
Scientific hypothesis. Deficiency of Drp1 in cerebellar PCs disrupts mitochondrial OXPHOS (including complexes III, IV, and V) and destabilizes the mitochondrial membrane. Reduced Coa6 expression further exacerbates complex IV impairment. These mitochondrial impairments culminate in a reduction of PC numbers, morphological abnormalities, and working memory deficits in mice. CoQ10 directly binds to Coa6 and elevates its expression, restoring complexes III, IV and V and mitochondrial membrane stability. Consequently, CoQ10 rescues the loss of dendritic spines in PCs and ameliorates working memory deficits in mice

In this study, we established PC-specific Drp1-deficient mouse models and summarized the major phenotypic changes observed in the cerebellum (Fig. 11). Drp1 deletion in PCs resulted in mitochondrial structural abnormalities, including disrupted cristae organization, reduced levels of mitochondrial respiratory chain complexes III, IV, and V, and decreased expression of Coa6 in cerebellar mitochondria. These mitochondrial alterations were associated with PC structural impairment and progressive deficits in cerebellar-dependent working memory. Following long-term CoQ10 administration (500 μM in drinking water for 75 days), several of these abnormalities were partially reversed. CoQ10 treatment was accompanied by increased Coa6 levels, recovery of mitochondrial ultrastructural features, restoration of respiratory chain complex levels (III–V), and attenuation of working memory deficits in Drp1-deficient mice. In addition, PC-specific up- or down-regulation of Coa6 modulated these mitochondrial and cellular phenotypes, and the beneficial effects of CoQ10 were no longer observed when Coa6 expression was knocked down.

CoQ10 is a lipid-soluble component of the mitochondrial ETC that plays a central role in cellular energy metabolism and mitochondrial homeostasis [36, 37]. Due to its involvement in electron transfer and redox balance, CoQ10 has been widely investigated in cardiovascular and neurological disorders characterized by mitochondrial dysfunction [38–40]. Previous studies have reported that CoQ10 supplementation can alleviate mitochondrial structural damage and neuronal degeneration in conditions involving cerebellar injury, including PC vulnerability [41]. In previous studies, CoQ10 was mainly used to treat diseases related to cognitive impairment by alleviating neuroinflammation [42, 43], inhibiting oxidative stress [44, 45] and restoring abnormal activity of acetylcholinesterase [46, 47]. Consistent with these observations, our results show that long-term CoQ10 supplementation in PC-Drp1^−/−^ mice is accompanied by partial improvement of mitochondrial ultrastructure, increased levels of respiratory chain complexes III, IV, and V, attenuation of PC structural deterioration, and improvement of cerebellar-dependent working memory. Notably, these improvements were observed in a developmental context of PC-specific Drp1 deletion, in which mitochondrial abnormalities emerge during postnatal maturation, prior to overt neuronal loss.

### Coa6 is directly targeted by CoQ10, mediating the protective effects of CoQ10 in PC-specific Drp1-deficient mice

Although CoQ10 has been extensively investigated in neurological disorders associated with mitochondrial dysfunction, most previous studies have evaluated its effects at the tissue or organismal level, without resolving the contribution of specific neuronal subtypes within vulnerable brain regions [36, 48]. In models of cognitive impairment and cerebellar injury, the reported benefits of CoQ10 supplementation have largely been attributed to global reductions in neuroinflammation, attenuation of oxidative stress, or modulation of neurotransmitter-related enzymes [49]. In the Drp1-deficient model first described by Kageyama et al., increased oxidative damage was identified as a key pathological mechanism. In that model, antioxidant treatments, including CoQ10, attenuated mitochondrial swelling and neuronal loss, indicating that oxidative stress is a major driver of pathology [50]. We emphasize that the Drp1-deficient model used in our study was intended to investigate how disturbances in mitochondrial dynamics affect neuronal function, rather than to replicate any single, specific neurodegenerative disease. However, while prior studies show that CoQ10 can ameliorate mitochondrial dysfunction and oxidative injury at the organism level, its specific molecular targets within vulnerable neurons remain unclear. In the present study, we addressed this gap in a PC-specific Drp1-deficient mouse model, in which mitochondrial abnormalities are restricted to a defined neuronal population during postnatal development. In this context, long-term CoQ10 supplementation was associated with partial restoration of respiratory chain complex levels, improvement of mitochondrial morphology, attenuation of oxidative stress, reduction of PC structural abnormalities, and improvement of cerebellar-dependent working memory performance. In conclusion, our model provides a basis for exploring the molecular mechanism by which CoQ10 directly affects the mitochondrial pathway in PCs.

Extensive prior research has conclusively demonstrated that CoQ10 exerts neuroprotective effects in various neurological disorders (such as AD, PD, depression, epilepsy, etc.) by serving as a soluble electron carrier in the mitochondrial ETC, scavenging ROS, and enhancing mitochondrial function [51, 52]. This has established the classic research paradigm of “CoQ10 = antioxidant + mitochondrial function support” [49]. However, in most studies, CoQ10 was used as a relatively diffuse antioxidant or electron carrier, with rare elucidation of its precise molecular targets at the level of specific mitochondrial protein assembly factors. Based on this, our study identified for the first time that Coa6 is the target molecule of CoQ10 through target capture. Thermal proteome profiling, CETSA, DARTS and surface plasmon resonance detection qualitatively and quantitatively demonstrated the stable binding between CoQ10 and Coa6. In contrast to previous findings, our study revealed—through molecular docking experiments—that CoQ10 exerts its effects in the cerebellum by binding to the mitochondrial cytochrome *c* oxidase assembly factor Coa6. Coa6 is a mitochondrial intermembrane space protein primarily involved in the assembly of complex IV of the respiratory chain [53]. Disruption of Coa6 impairs complex IV assembly and activity, and can lead to combined deficiencies in complexes I and IV due to defective respiratory chain biogenesis [35]. Unlike traditional research that regards CoQ10 as a "broad-spectrum mitochondrial protective agent", we propose a targeted respiratory chain assembly mechanism with Coa6 as the key assembly node: CoQ10 not only participates in electron transport, but also can reshape the respiratory chain function at the assembly level by precisely regulating the key assembly factor Coa6. In addition, our study showed that CoQ10 increased the levels of complexes III and V in the cerebellar mitochondrial respiratory chain. Therefore, we hypothesize that CoQ10 may attenuate the Drp1 deficieny-induced working memory impairment by enhancing the activities of complexes III, IV, and V via interacting with Coa6. To directly test whether Coa6 is a key mediator of the effects of CoQ10, we manipulated Coa6 expression in PCs using viral vectors in the absence of exogenous CoQ10 treatment. Viral overexpression of Coa6 in Drp1-deficient PCs partially recapitulated the improvements in mitochondrial respiratory chain organization and working memory performance observed with CoQ10 supplementation, indicating that increased Coa6 levels alone can mimic the effects of CoQ10 on ETC integrity. In contrast, knockdown of Coa6 exacerbated mitochondrial dysfunction and working memory deficits, further supporting the hypothesis that Coa6 plays an essential role in the mechanistic pathway through which CoQ10 acts. These results suggest that in this model, Coa6 improves the assembly of mitochondrial respiratory chain complexes as well as their stability, which in turn supports electron transport and energy production.

### Specific CoQ10 binding to Coa6 at the K18 residue may have driven coordinated increases of activities of mitochondrial respiratory chain complexes

In our Drp1-deficient model, molecular docking illustrated that the methoxy group of CoQ10 formed hydrogen bonds exclusively with the 18th lysine residue (K18) of Coa6, suggesting a highly specific interaction. Previous studies indicate that when CoQ10 binds to proteins, the hydrogen bonds are usually involved in conjunction with hydrophobic interactions, enhancing the stability and specificity of the complexes [54, 55], which aligns with our results. Moreover, the cerebellar expression levels of Coa6 significantly increased following CoQ10 supplementation. This may result from CoQ10-induced promotion of Coa6 biogenesis or Coa6 stabilization through inhibited degradation. Either mechanism would be expected to enhance mitochondrial complex IV activity (as supported by increased COX4 levels), reduce ROS production, and restore neuronal energy metabolism. Although previous studies demonstrated that CoQ10 increases the levels of complexes I, II, and IV [42], restores the decreased complex IV activity and complex IV-OPA1 binding in aged mice [56], enhances activities of complexes I/II/III in Parkinson’s disease [57], and modulates complexes II/III in Huntington’s disease [58], the precise mechanisms underlying CoQ10-induced enhancement of complex I, II, III, and V functions remain incompletely understood. Based on our findings, we propose two potential mechanisms: (1) CoQ10 enhances the stability of Coa6 (a complex IV assembly factor), which may subsequently promote the function or stability of complexes III and V through indirect effects on overall respiratory chain integrity or supercomplex formation; and (2) the improved complex IV activity caused by CoQ10 subsequently promotes the activities of complexes III and V. Crucially, using a cerebellar injury model, our study systematically demonstrated that CoQ10 can simultaneously enhance the functions of complexes III, IV, and V.

### Loss of Drp1 reduces the stability of Coa6 at the protein level

Although we demonstrate that CoQ10 directly binds Coa6 and restores mitochondrial function in Drp1-deficient PCs, the direct molecular link between Drp1 deficiency and Coa6 downregulation remains to be fully defined. Our immunofluorescence data showed a marked reduction of Coa6 protein in Drp1-deficient PCs, whereas qPCR analysis indicated that Coa6 mRNA levels were not significantly changed. To investigate whether Coa6 stability was affected, we performed tissue-CETSA on cerebellar lysates. In Drp1-deficient tissue, Coa6 exhibited a shift toward earlier loss of solubility during thermal stress, consistent with reduced thermal stability and an increased propensity for denaturation or degradation compared with control tissue. This shift reflects a reduction in thermal stability of Coa6 in the absence of Drp1, consistent with a protein that is more prone to unfolding and loss of its native structure when the temperature increases. This observation supports the idea that Drp1 loss may compromise the post-translational stability or turnover of Coa6 rather than its gene expression. Coa6 is a protein located in the mitochondrial intermembrane space that is required for the proper assembly of cytochrome *c* oxidase (also called complex IV) in the respiratory chain. During the formation of complex IV, COA6 helps coordinate the incorporation of copper into the COX2 subunit, which is essential for complex IV to function. Mutations in Coa6 that disrupt its maturation or stability—even if they do not prevent Coa6 from entering mitochondria—lead to defects in complex IV assembly and reduced enzyme activity [59]. Although the exact molecular events connecting Drp1-mediated mitochondrial dynamics and Coa6 stability are not yet defined, loss of Drp1 disrupts mitochondrial morphology and protein homeostasis, which may in turn reduce the stability of assembly factors such as Coa6, making them more prone to misfolding and degradation. Notably, viral modulation of Coa6 expression did not alter Drp1 levels, supporting the notion that Coa6 downregulation is a downstream consequence, not a driver, of Drp1 loss. Although the precise molecular basis remains unclear, our data suggest that Drp1 deficiency may alter the mitochondrial protein quality control environment, leading to reduced stability of Coa6 protein rather than changes in its transcription.

### PC dendritic abnormalities and cell loss occur in Drp1-deficient mice and their order of occurrence remain unclear

Taken together with previous evidence in similar models that the progressive loss of PCs reflects neuronal death rather than mere loss of cell identity or downregulation of specific markers [60], we interpret the reduction in PC number as an index of neuronal loss rather than downregulation of specific markers. Based on the current dataset, we can reliably conclude that PC dendritic pathology (including reduced dendritic complexity and spine alterations) and decreases in PC number co-occur in Drp1-deficient mice and are both correlated with working memory impairment. Changes in the dendritic architecture and spine structure of PCs are expected to disrupt synaptic input integration and neuronal output, given the extensive branching and high density of synaptic contacts these neurons normally possess. However, the available data do not allow us to determine whether the dendritic structural changes precede cell loss, whether one form of pathology causally drives the other, or whether either alone is sufficient to cause the working memory deficits. This limitation reflects the intertwined nature of morphological degeneration and cell loss in degenerative conditions, in which structural remodeling, synaptic dysfunction, and cell death often proceed in parallel rather than as isolated events, making it challenging to segregate their individual contributions. Future work using longitudinal designs or targeted interventions will be required to clarify the temporal sequence and causal relationships among dendritic degeneration, PC loss, and cognitive dysfunction.

### Drp1-deficient mice show age-dependent working-memory decline in the eight-arm maze, which suggests the need of early intervention

Considering that the Drp1-deficient mice used in our experiments have ataxia [50, 60], which may interfere with the results of the water maze, we used the eight-arm maze test to explore working memory impairment in mice. The eight-arm maze is a classic behavioral tool for evaluating working memory and spatial learning ability, which is widely used in neuroscience and pharmacology research. Its design allows simultaneous measurement of working memory and reference memory, with high sensitivity and repeatability, and is considered one of the reliable methods for studying working memory [61, 62]. Importantly, all of our conclusions regarding working memory deficits are strictly based on the eight-arm maze performance. Other behavioral assays were used for distinct purposes: the Morris water maze was used only to describe the overall trajectory of cognitive decline with age in the Drp1-deficient model, the rotarod test was used to assess motor coordination, and the open field test was used to evaluate spontaneous locomotor activity and anxiety-like behavior. In our model, working memory performance deteriorated with age in Drp1-deficient mice, while the control mice consistently made few or no working memory errors. The Drp1-deficient mice began to show an increase in errors at 1 and 2 months of age. However, these early increases did not reach statistical significance in two-factor repeated measures ANOVA. By 3 months of age, the Drp1-deficient mice exhibited pronounced working memory impairment, indicating a robust deficit in maintaining and updating spatial information. When CoQ10 was administered from postnatal day 15, the PC-Drp1^−/−^ mice showed significant improvement in working memory deficits compared to controls. Previous studies have reported that Drp1 plays a key role in PC and cerebellar development, as its knockdown results in a 40% reduction in cerebellar volume and mitochondrial abnormalities [23]. Our previous study showed that although mitochondrial transplantation in juvenile mice ameliorated motor dysfunction caused by Drp1 deficiency, Drp1 upregulation in PCs at 1 month of age or mitochondria transplantation in adult mice failed to improve ataxia [60]. Consistent with previous studies, our findings highlight an important role for Drp1 in PC development.

### Drp1 loss during cerebellar development drives the phenotypes in our model and whether the same deficits would occur after adult-onset Drp1 loss remains to be tested

In our model, deletion of Drp1 in PCs occurred during embryogenesis. The resulting deficits in PC integrity and working memory are highly dependent on the developmental stage. Previous work has shown that deletion of Drp1 in post-mitotic cerebellar PCs during early development leads to oxidative damage, disrupted mitochondrial morphology, and progressive PC degeneration, indicating that mitochondrial division is critical for neuronal survival in this period [50]. Together, these findings imply that PCs and related cognitive circuits are particularly vulnerable to Drp1 loss during stages when mitochondrial dynamics is critical for dendritic growth, synaptogenesis, and early metabolic adaptation. Therefore, the pathological and behavioral phenotypes observed in our PC-specific Drp1-KO mice should be interpreted in the context of disrupted mitochondrial dynamics during critical periods of cerebellar maturation. It remains unknown whether Drp1 KO in cerebellar PCs of adult mice may lead to similar neuronal and motor function deficits, which requires further investigation.

### CoQ10 crosses the blood–brain barrier (BBB) in mice but whether it does so in adults still needs further study

Administration of CoQ10 in mice for 75 days can increase CoQ10 levels in cerebellar tissue, suggesting that achieving central exposure in rodent models is feasible. In clinical studies, several trials have shown that high-dose CoQ10 or ubiquinol can significantly increase plasma CoQ10 levels, and some of these studies have also reported increased CoQ10 concentrations in cerebrospinal fluid [63–65]. However, direct evidence for CoQ10 crossing the BBB in humans is still limited. To enhance the translational relevance, two strategies could be considered. First, cerebrospinal fluid CoQ10 levels could be measured using highly sensitive analytical methods such as LC–MS/MS, and used as a surrogate marker of central exposure; previous work has suggested that cerebrospinal fluid assessment is a reasonable approach to evaluate CoQ10 status in the nervous system [66]. Second, high-bioavailability formulations, including lipid-based carriers, nano-formulations, liposomal preparations, or emulsified CoQ10, could be explored to improve oral absorption and potentially enhance BBB penetration [67].

### Limitations

Several limitations should be considered. First, all data were derived from preclinical mouse models and molecular/biochemical assays, so the identified pathway cannot yet be regarded as a clinically validated therapeutic target. Second, we focused primarily on mechanistic elucidation of the effects of CoQ10 on mitochondrial function and behavioral phenotypes. Systematic body weight measurements or water intake records were not included in this study. As a result, we were unable to calculate the actual daily dose of CoQ10 in terms of mg/kg per day for individual animals, and could not perform standard bovine serum albumin-based conversions to estimate human equivalent doses. Consequently, our conclusions are confined to the mechanistic effects observed in the mouse model and should not be interpreted suggestive for human supplement doses or clinical recommendations. Third, because CoQ10 is a highly hydrophobic molecule that is difficult to label without altering its properties, we were unable to perform direct in vivo pull-down or fluorescence-tracking experiments. Thus we relied on indirect functional and biochemical readouts rather than direct visualization of CoQ10 distribution. Fourth, the CoQ10–Coa6 pathway was identified and functionally validated specifically in the cerebellar PC-specific Drp1-deficient model. Its relevance to other neuronal populations, brain regions, or disease contexts remains to be determined. Finally, although metabolic cage data were normal, we cannot exclude subtle effects of 3% DMSO on neurons, mitochondria, or cellular ultrastructure. Future work is needed to explore alternative solvents or delivery methods to minimize potential vehicle effects [68].

## Supplementary Information

Additional file 1. **Fig. S1**. Flowchart of network pharmacology.** Fig. S2**. Drp1 expression in different cerebellar cells of PC-Drp1^-/-^ mice. **Fig. S3**. Comparison of PCs and behaviors of control mice from 1 to 3 months old. **Fig. S4**. PSD95 immunofluorescence staining. **Fig. S5**. OCR, ECAR and FAO detection of the cerebellum after PC-Drp1^-/-^ and CoQ10 intervention. **Fig. S6**. Metabolic cage measurements in PC-specific Drp1-deficient mice under different interventions. **Fig. S7**. Detection of CoQ10 content in the cerebellum and serum. **Fig. S8**. The content of CoQ10 and the stability of cerebellar Coa6 in mice after CoQ10 intervention. **Fig. S9**. Comparison after intervention with different concentrations of CoQ10. **Fig. S10**. Electrophysiological detection of PCs after CoQ10 intervention. **Fig. S11** Detection of the relationship between Drp1 and Coa6.

Additional file 2. Full uncropped blots.

## Data Availability

The datasets produced and analysed during the current study are available from the corresponding authors on reasonable request.

## Abbreviations

AAVs: Adeno-associated viruses
AD: Alzheimer's disease
ATP: Adenosine triphosphate
BBB: Blood–brain barrier
CETSA: Cellular thermal shift assay
Coa6: Cytochrome c oxidase assembly factor 6
CoQ10: Coenzyme Q10
COX4: Cytochrome c oxidase 4
DARTS: Drug affinity responsive target stability
DMSO: Dimethyl sulfoxide
Drp1: Dynamin-related protein 1
ECAR: Extracellular acidification rate
ETC: Electron transport chain
GFP: Green fluorescent protein
GO: Gene Ontology
GO-BP: GO biological processes
GO-CC: GO cellular components
GO-MF: GO molecular function
GPx1: Glutathione peroxidase 1
HCN: Hyperpolarization activated cyclic nucleotide gated channels
HD: Huntington's disease
KEGG: Kyoto Encyclopedia of Genes and Genomes
Mb: Myoglobin
MiNA: Mitochondrial network analysis
MWM: Morris water maze
MMP: Mitochondrial membrane potential
OXPHOS: Oxidative phosphorylation
PC: Purkinje cell
PCR: Polymerase chain reaction
PD: Parkinson's disease
PSD95: Postsynaptic density protein 95
RER: Respiratory exchange ratio
ROS: Reactive oxygen species
SOD1: Superoxide dismutase
TEM: Transmission electron microscope
