# Supplementary material for: The Drp1-CoQ10-Coa6-ETC axis represents a therapeutic potential for working memory impairment caused by neuronal mitochondrial dysfunction
Source: Transl Neurodegener. 2026 Apr 27;15:18. doi: 10.1186/s40035-026-00552-6 (PMC13112672; doi:10.1186/s40035-026-00552-6)
Supplement: Supplementary file 1 — Additional file 1. Fig. S1. Flowchart of network pharmacology. Fig. S2. Drp1 expression in different cerebellar cells of PC-Drp1-/- mice. Fig. S3. Comparison of PCs and behaviors of control mice from 1 to 3 months old. Fig. S4. PSD95 immunofluorescence staining. Fig. S5. OCR, ECAR and FAO detection of the cerebellum after PC-Drp1-/- and CoQ10 intervention. Fig. S6. Metabolic cage measurements in PC-specific Drp1-deficient mice under different interventions. Fig. S7. Detection of CoQ10 content in the cerebellum and serum. Fig. S8. The content of CoQ10 and the stability of cerebellar Coa6 in mice after CoQ10 intervention. Fig. S9. Comparison after intervention with different concentrations of CoQ10. Fig. S10. Electrophysiological detection of PCs after CoQ10 intervention. Fig. S11 Detection of the relationship between Drp1 and Coa6. [file 40035_2026_552_MOESM1_ESM.docx]

**
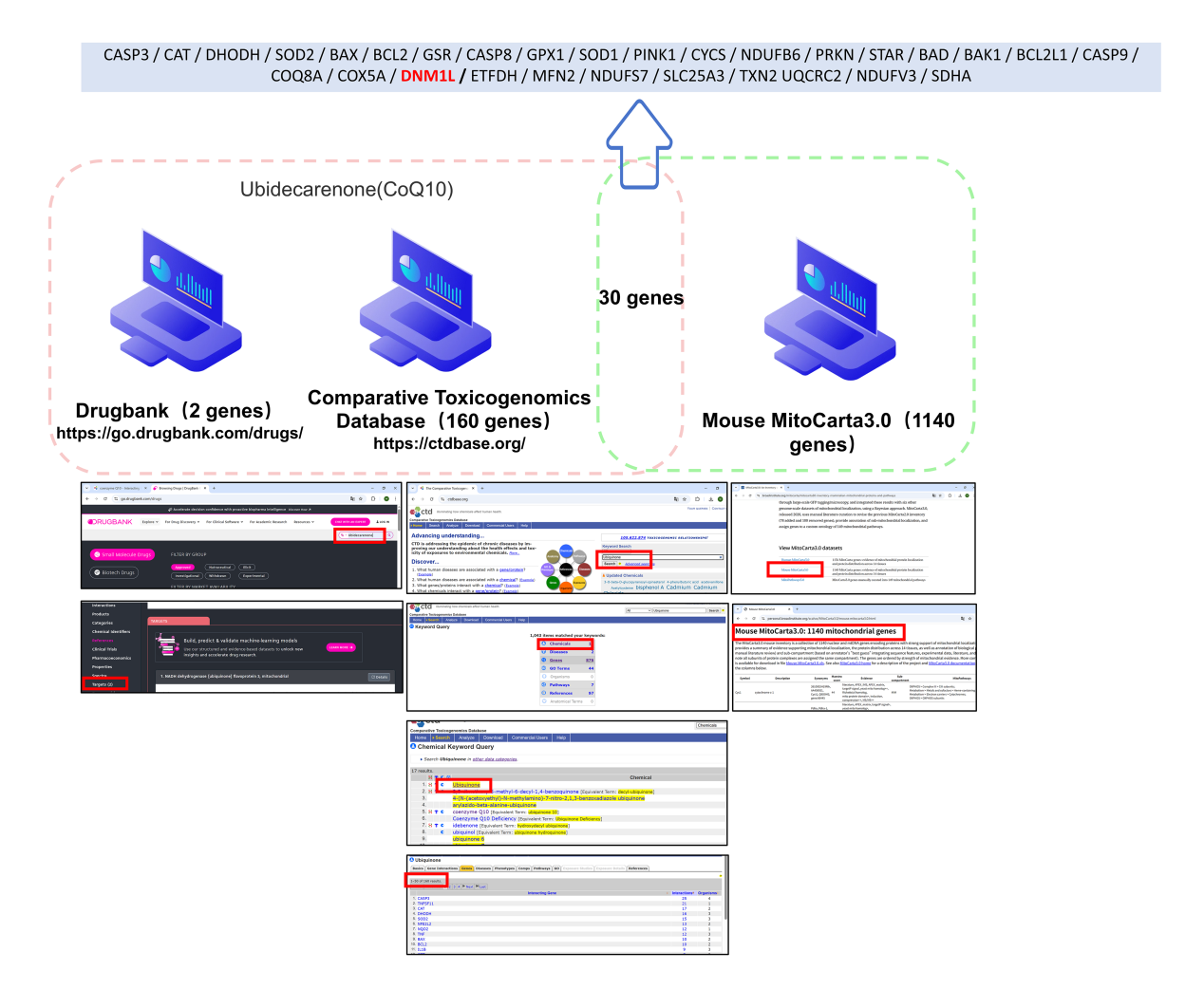
**

**Figure S1 Flowchart of network pharmacology.** Three websites were used to explore the intersection of CoQ10 and mitochondria.


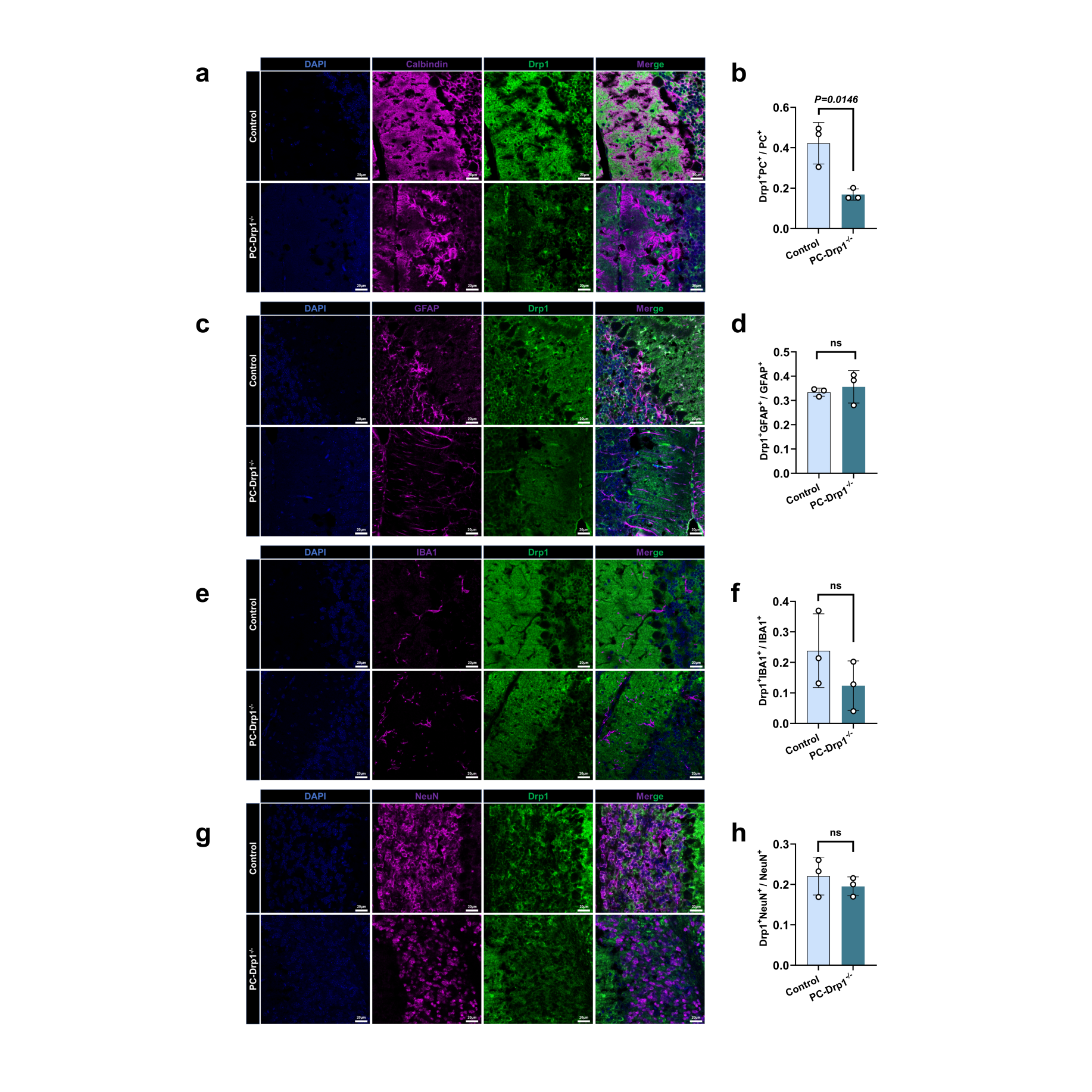


**Figure S2 Drp1 expression in different cerebellar cells of PC-Drp1^-/-^ mice.** **a** The representative images show the co-localization of Drp1 and Calbindin (Purkinje cells marker) in 3M PC-Drp1^-/-^ and control mouse. Bar = 20 μm, *n* = 3 mice. **b** Quantitative analysis of (**a**). **c** The representative images show the co-localization of Drp1 and GFAP (astrocytes marker) in 3M PC-Drp1^-/-^ and control mouse. Bar = 20 μm, *n* = 3 mice. **d** Quantitative analysis of (**c**). **e** The representative images show the co-localization of Drp1 and Iba1 (microglia marker) in 3M PC-Drp1^-/-^ and control mouse. Bar = 20 μm, *n* = 3 mice. **f** Quantitative analysis of (**e**).**g** The representative images show the co-localization of Drp1 and NeuN (Neuron marker) in 3M PC-Drp1^-/-^ and control mouse. Bar = 20 μm, *n* = 3 mice. **h** Quantitative analysis of (**g**).The data were presented as means ± SD. *P* were calculated by unpaired two-tailed t-test (**b**, **d**, **f** and **h**). ns, no significance.

**
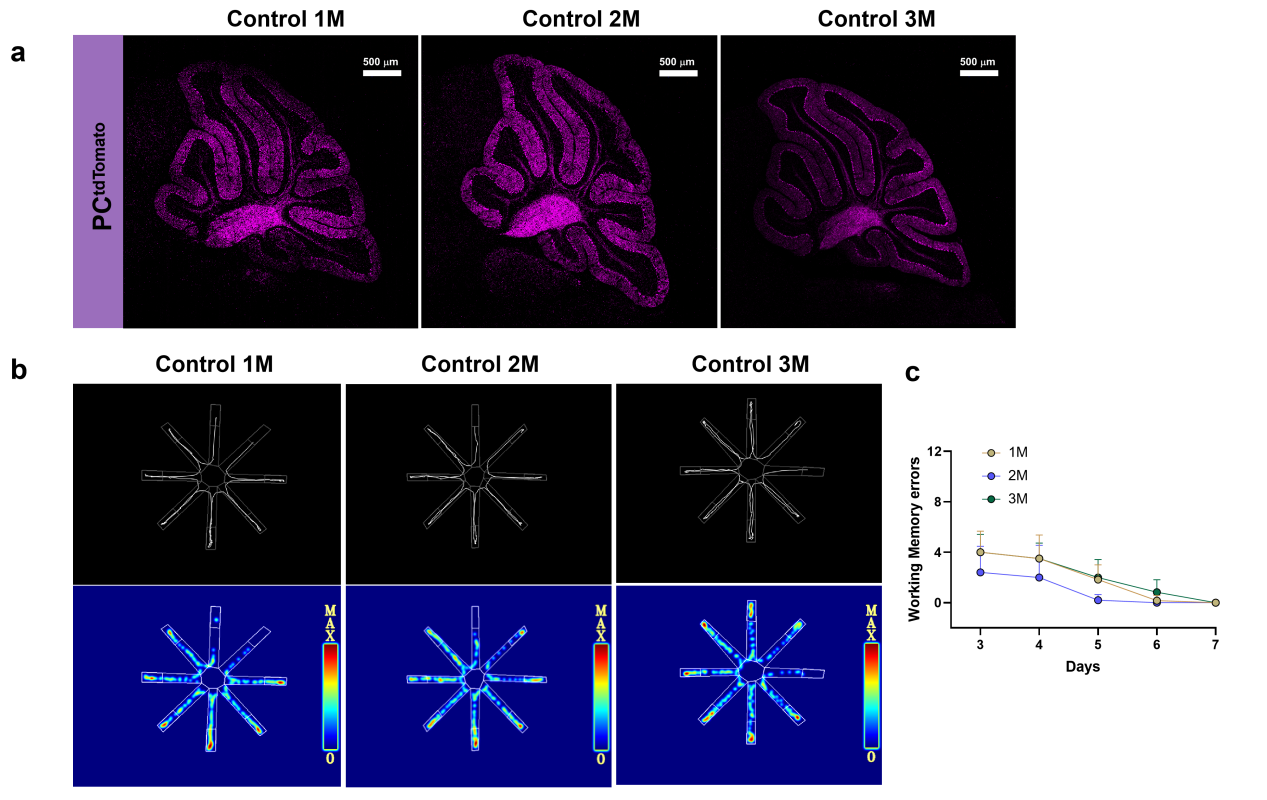
**

**Figure S3 Comparison of PCs and behaviors of control mice from 1 to 3 months old.** **a** Sagittal representation of PCs (PC^tdTomato^) in 1-3 month-old control mice. Bar = 500 μm. **b,c** Representative diagrams and quantitative analysis of the eight-arm maze experiment in 1-3 month-old control mice. *n* = 6 mice.The data were presented as means ± SD. *P* were calculated by two-way ANOVA (**c**).

**
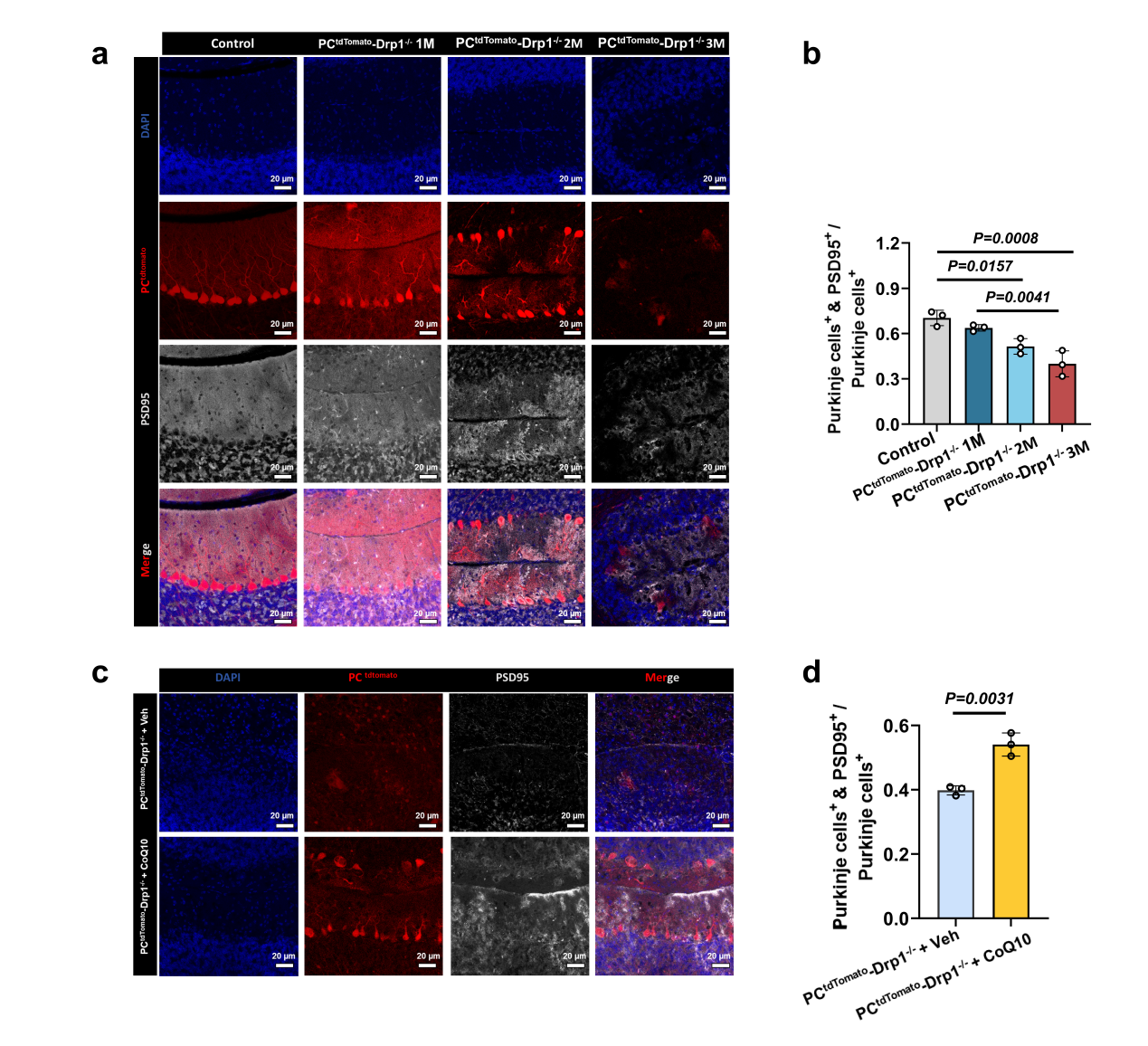
**

**Figure S4 PSD95 immunofluorescence staining.** **a** Representative graph of PSD95 expression in PCs of 1 - 3 month-old PC^tdTomato^-Drp1^-/-^ mice. Bar = 20 μm, *n* = 3 mice. **b** Quantitative analysis of (**a**). **c** Representative graph of PSD95 expression in PC^tdTomato^-Drp1^-/-^ + Veh and PC^tdTomato^-Drp1^-/-^ + CoQ10 mice. Bar = 20 μm, *n* = 3 mice. **d** Quantitative analysis of (**c**).The data were presented as means ± SD. *P* were calculated by one-way ANOVA (**b**) and unpaired two-tailed t-test (**d**).

**
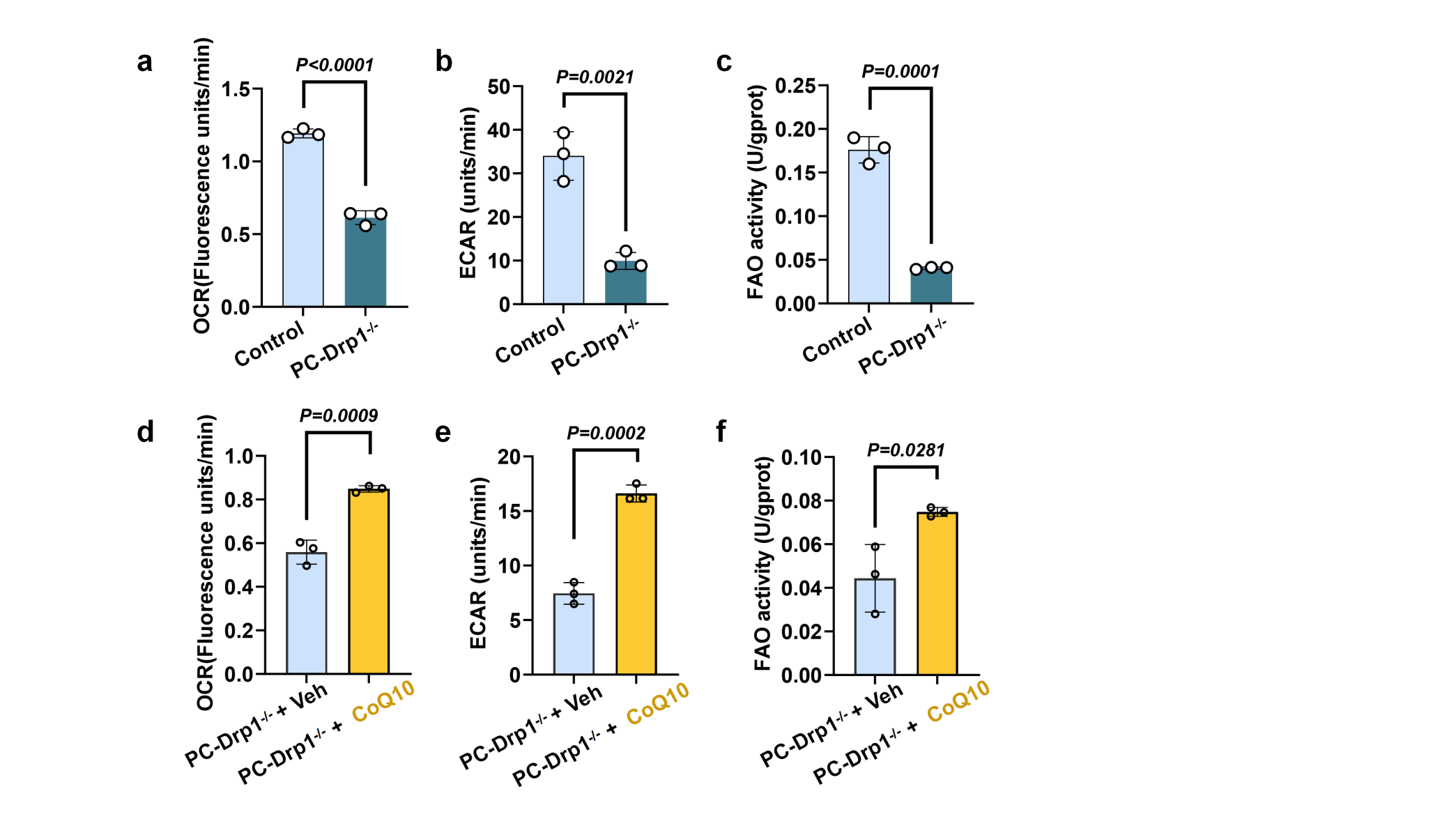
**

**Figure S5 OCR, ECAR and FAO detection of the cerebellum after PC-Drp1^-/-^ and CoQ10 intervention. a, b** and **c** Quantitative analysis of OCR, ECAR and FAO detection of Control and PC-Drp1^-/-^. *n* = 3 mice. **d, e** and **f** Quantitative analysis of OCR, ECAR and FAO detection of PC-Drp1^-/-^ + Veh and PC-Drp1^-/-^+ CoQ10. *n* = 3 mice. The data were presented as means ± SD. *P* were calculated by unpaired two-tailed t-test (**a-f**).

**
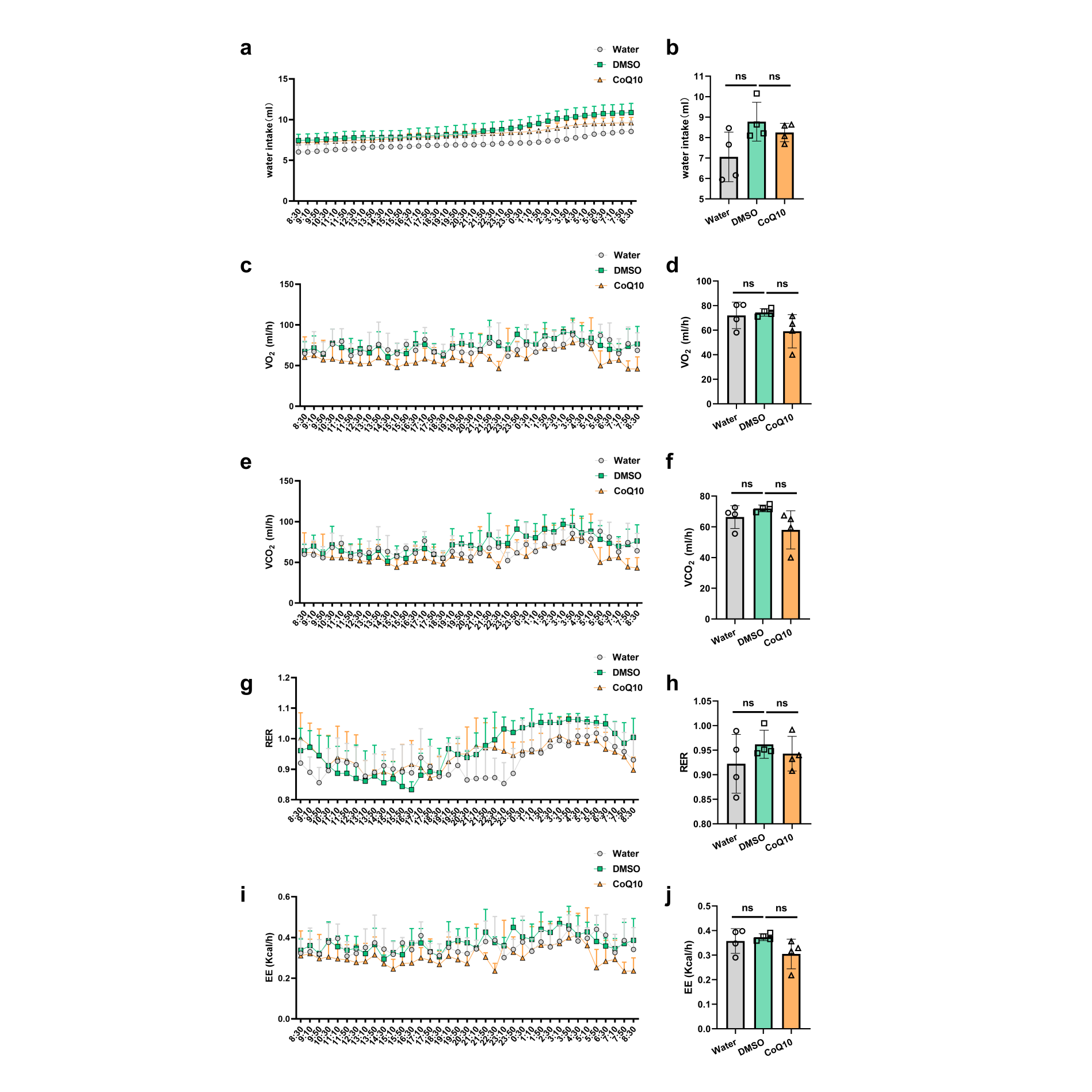
**

**Figure S6 Metabolic cage measurements in PC-specific Drp1-deficient mice under different interventions. a-j** Quantitative analysis of the 24-hour changes of water intake, VO_2_, VCO_2_, RER and EE in the metabolic cages of PC-Drp1^-/-^ mice after drinking normal water, 3% DMSO and CoQ10 respectively, as well as the average values. *n* = 4 mice. The data were presented as means ± SD. *P* were calculated by one-way ANOVA (**b, d, f, h** and **j**). ns, no significance.

**
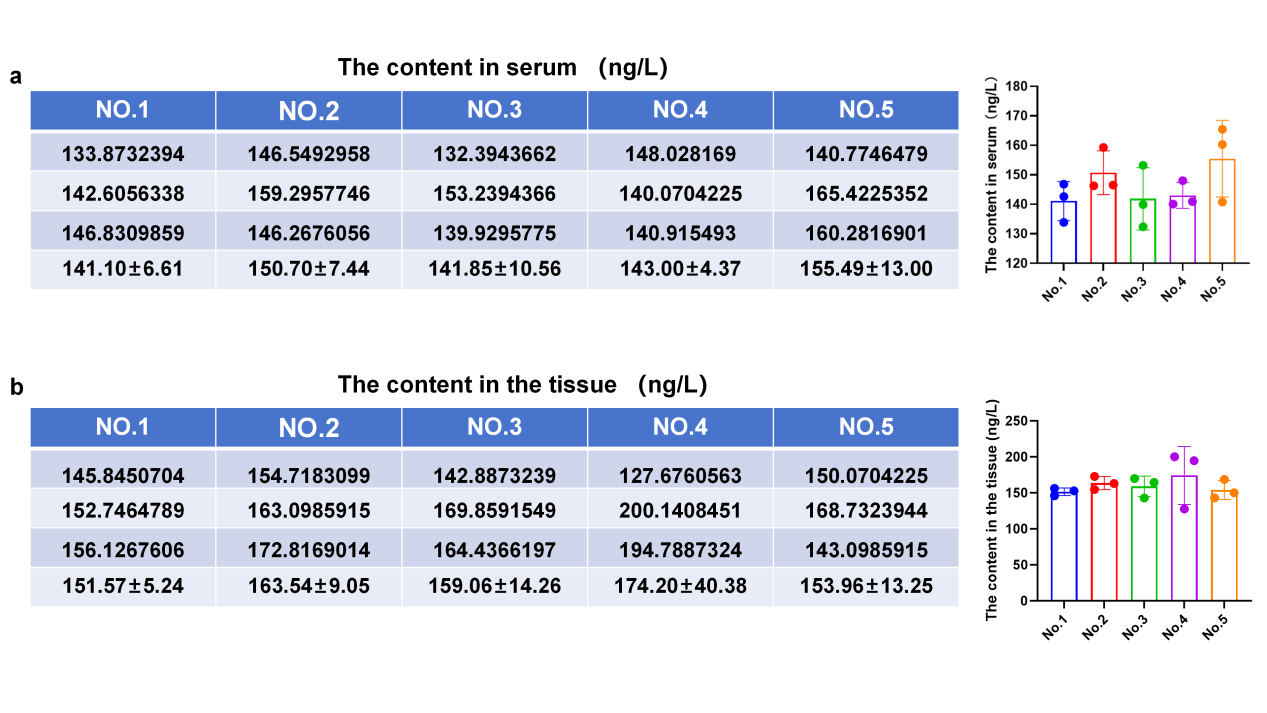
**

**Figure S7 Detection of CoQ10 content in the cerebellum and serum.** **a** The content value of CoQ10 in the serum of 5 PC-Drp1^-/-^ mice fed in a cage (1 mouse; 3 technical replicates). **b** The content value of CoQ10 in the cerebellum of 5 PC-Drp1^-/-^ mice fed in a cage (1 mouse; 3 technical replicates).

**
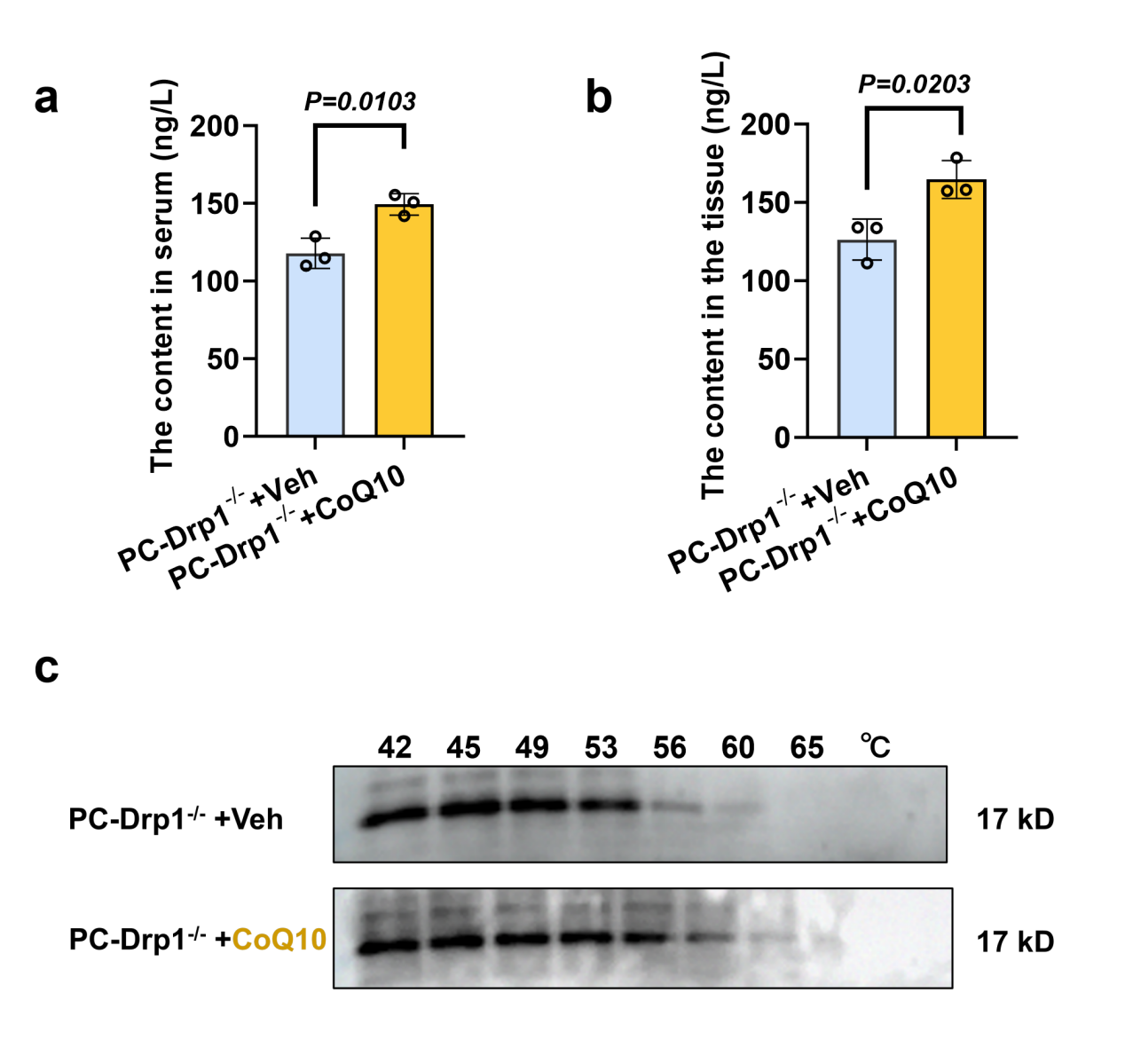
**

**Figure S8 The content of CoQ10 and the stability of cerebellar Coa6 in mice after CoQ10 intervention. a** Quantitative detection of CoQ10 in the serum of mice. *n* = 3 mice. **b** Quantitative detection of CoQ10 in the cerebellum of mice. *n* = 3 mice.

**c** Tissue CETSA was used to detect the stability of Coa6 after CoQ10 intervention. *n* = 3 mice. The data were presented as means ± SD. *P* were calculated by unpaired two-tailed t-test (**a** and **b**).


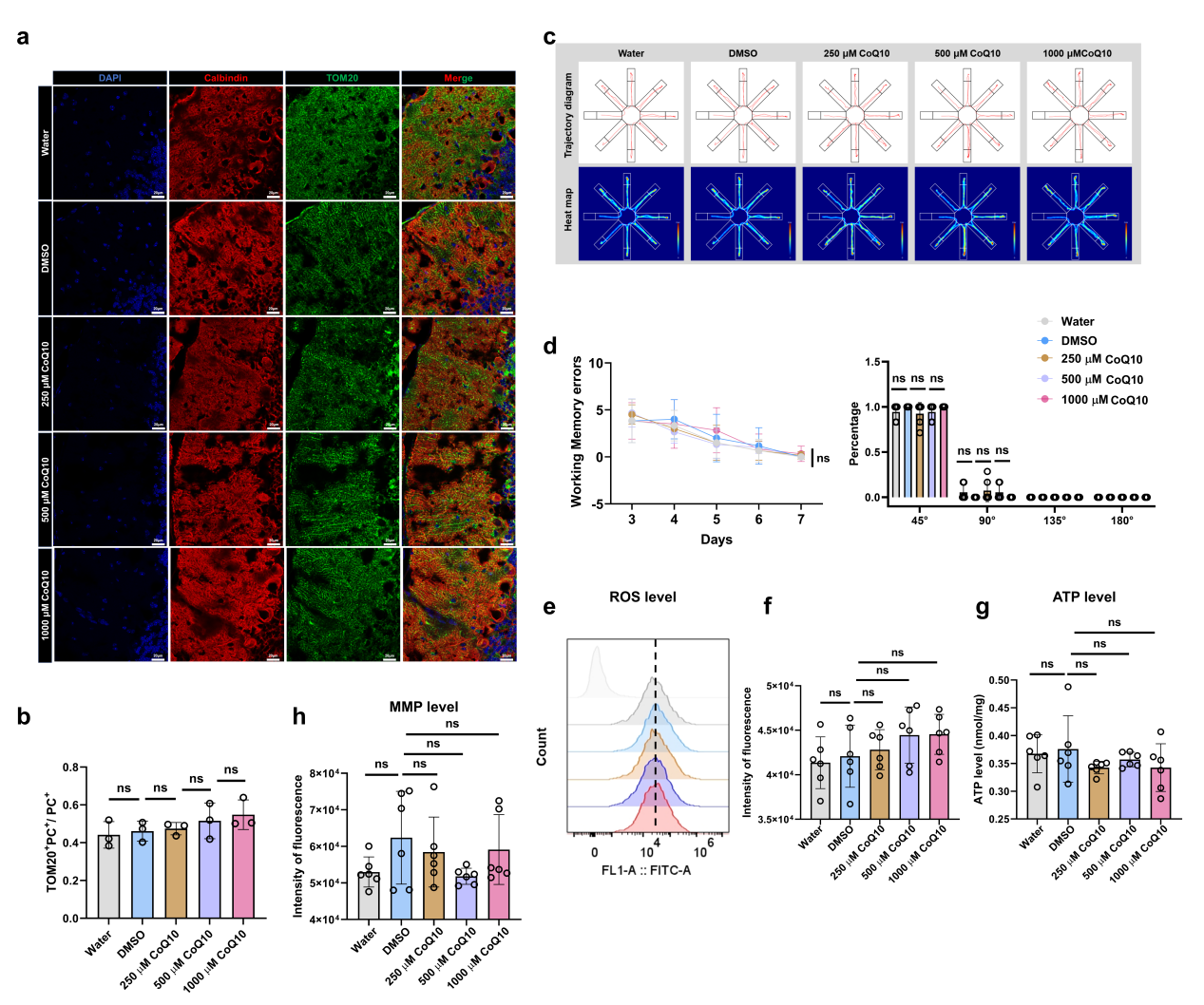


**Figure S9 Comparison after intervention with different concentrations of CoQ10. a** Representative diagrams of PCs and mitochondria in normal mice after intervention with different concentrations of CoQ10. Bar = 20 μm. **b** Quantitative analysis of (**a**), *n* = 3 mice. **c,d** Representative diagram and quantitative analysis of the eight-arm maze experiment. *n* = 6 mice. **e,f** Flow cytometry representative diagram and quantitative analysis of ROS activity detected by DCFH-DA. *n* = 6 mice. **g** Detection of ATP activity. *n* = 6 mice. **h** JC-1 detects mitochondrial membrane potential. *n* = 6 mice. The data were presented as means ± SD. *P* were calculated by one-way ANOVA (**b, f, g** and **h**) or two-way ANOVA (**d**). ns, no significance.

**
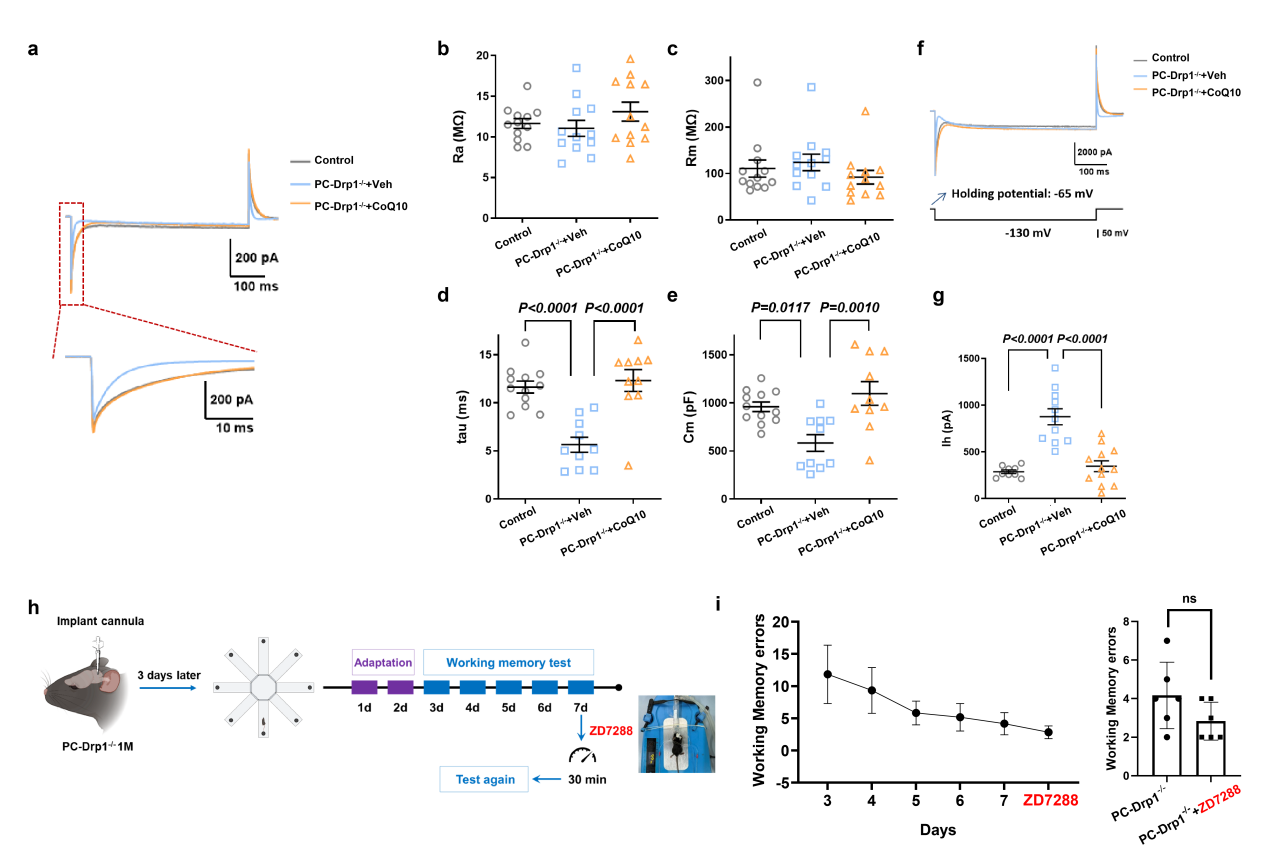
**

**Figure S10 Electrophysiological detection of PCs after CoQ10 intervention. a** Patch clamp electrophysiologic recording of control, PC-Drp1^-/-^ + Veh mice, and PC-Drp1^-/-^ + CoQ10 mice. **b-e** Quantitative analysis of Ra (**b**), Rm (**c**), tau (**d**), and Cm (**e**), *n* = 12 neurons. **f** Patch clamp electrophysiologic recording at voltage-clamp mode with the holding potential of - 65 mV of PC-Drp1^-/-^ + Veh mice, and PC-Drp1^-/-^ + CoQ10 mice. **g** Quantitative analysis of Ih, *n* = 12 neurons. **h** ZD7288 tubal administration. **i** Working memory was detected using an eight-arm maze after ZD7288 intervention. *n* = 6 mice. The data were presented as means ± SD. *P* were calculated by one-way ANOVA (**b, c, d, e, g**) or unpaired two-tailed t-test (**i**). ns, no significance.





**Figure S11 Detection of the relationship between Drp1 and Coa6. a** Representative graph and quantitative analysis of Coa6 expression in PC-Drp1^-/-^ mouse PCs. Bar = 20 μm. **b** Quantitative analysis of (**a**), *n* = 3 mice. **c** Quantitative analysis of Coa6 mRNA by qPCR. *n* = 3 mice. **d** Tissue CETSA was used to detect the stability of Coa6 in PC-Drp1^-/-^ mouse. *n* = 3 mice. **e,f** Representative graphs and quantitative analysis of Drp1 expression in PCs after down-regulation of Coa6 virus by immunofluorescence detection. Bar = 20 μm, *n* = 3 mice. **g,h** Representative graphs and quantitative analysis of Drp1 expression in PCs after upregulation of Coa6 virus by immunofluorescence detection. Bar = 20 μm, *n* = 3 mice. The data were presented as means ± SD. *P* were calculated by unpaired two-tailed t-test (**b, c, f** and **h**). ns, no significance.

| **Table S1. The primers used in gene identification.** | |  |
| --- | --- | --- |
| **Gene** | **Sequence (5' to 3')** | **Length** |
| Drp1 ^flox/flox^ | F: CCACTTTAGGTACTCATAACACA | Mutant: 292 bp; WT: 231 bp |
|  | R: ATTGTCATACATTCAGATAGGG |  |
| Pcp2-Cre | F: CACCAGTCTTAGTTACACAAATG | Mutant: 576 bp; WT: - |
|  | R: GGACAGGTAATGGTTGTCTGG |  |
| Mito GFP | F1: CCCAAAGTCGCTCTGAGTTGTTA | Mutant: 375 bp; WT: 426 bp |
|  | R1: TCGGGTGAGCATGTCTTTAATCT |  |
|  | R3: TGGCGTTACTATGGGAACATACGTC |  |
| B6-G/R | F: TACGGCATGGACGAGCTGTAC | Mutant: 1465 bp; WT: - |
|  | R: CCAACCTTTGTTCATGGCAG |  |
