# Supplementary material for: The Drp1-CoQ10-Coa6-ETC axis represents a therapeutic potential for working memory impairment caused by neuronal mitochondrial dysfunction
Source: Transl Neurodegener. 2026 Apr 27;15:18. doi: 10.1186/s40035-026-00552-6 (PMC13112672; doi:10.1186/s40035-026-00552-6)

Figure 2c

Drp1 (Control vs. PC-Drp1$^{-/-}$)

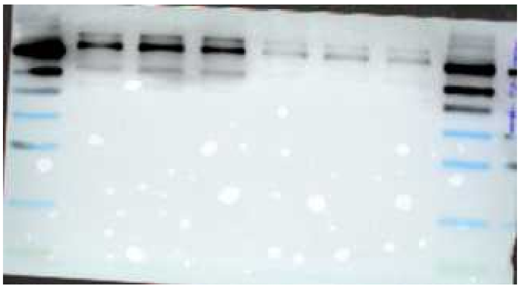

β-actin (Control vs. PC-Drp1$^{-/-}$)

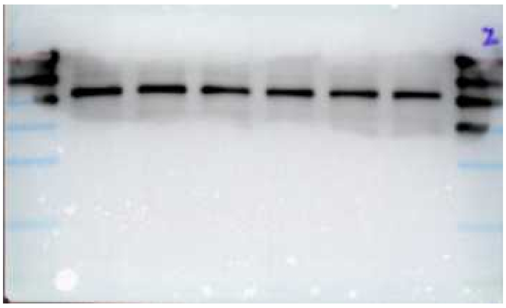

Figure 4p

SOD1 (Control vs. PC-Drp1$^{-/-}$), the lower strip in the blot

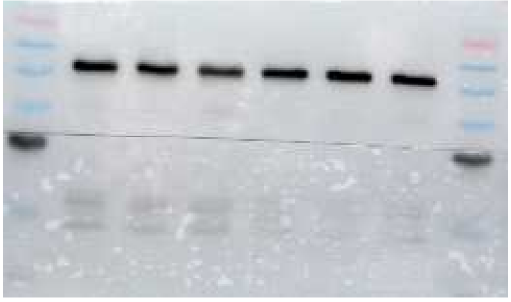

β-actin (Control vs. PC-Drp1$^{-/-}$), the upper strip in the blot

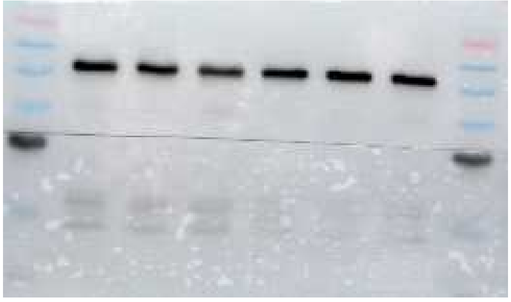

Figure 4q

GPx1 (Control vs. PC-Drp1$^{-/-}$)

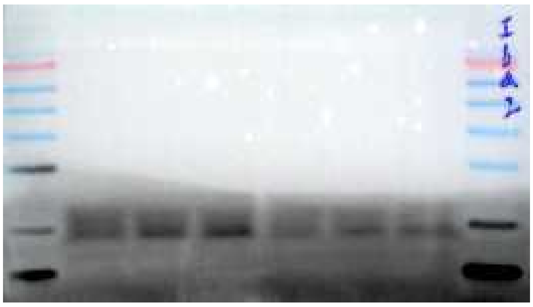

β-actin (Control vs. PC-Drp1$^{-/-}$)

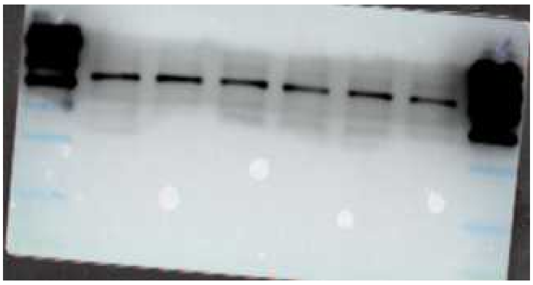

Figure 4r

CI-V (Control vs. PC-Drp1$^{-/-}$)

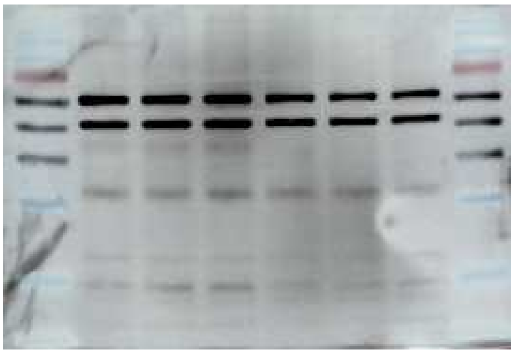

β-actin (Control vs. PC-Drp1$^{-/-}$)

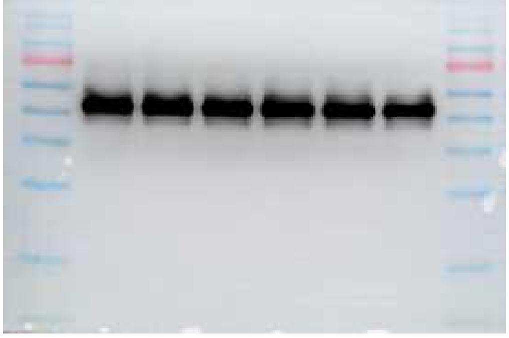

Figure 5f

IBA1 (Control vs. PC-Drp1$^{-/-}$)

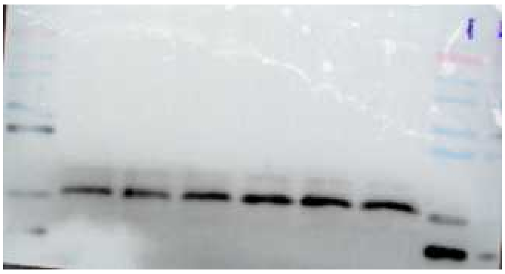

β-actin (Control vs. PC-Drp1$^{-/-}$)

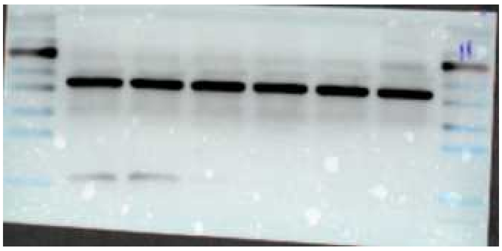

Figure 7i

Coa6 (Control vs. PC-Drp1$^{-/-}$ + Veh vs. PC-Drp1$^{-/-}$ + CoQ10)

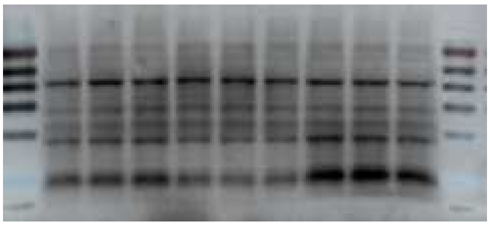

β-actin (Control vs. PC-Drp1$^{-/-}$ + Veh vs. PC-Drp1$^{-/-}$ + CoQ10)

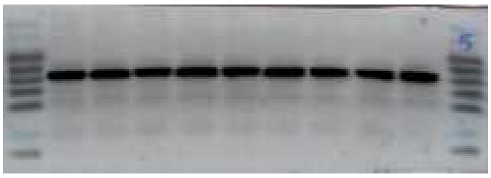

Figure 8f

CI-V (PC-Drp1$^{-/-}$ + Veh vs. PC-Drp1$^{-/-}$ + CoQ10)

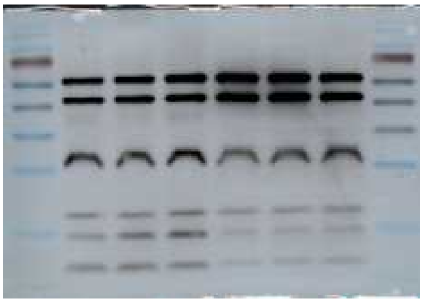

β-actin (PC-Drp1$^{-/-}$ + Veh vs. PC-Drp1$^{-/-}$ + CoQ10)

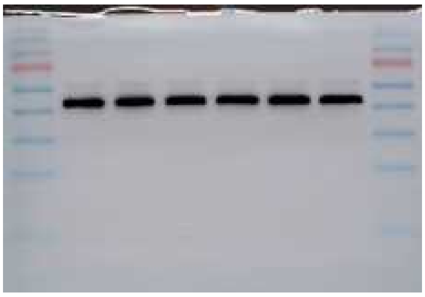

Figure 8g

COX4 (Control vs. PC-Drp1$^{-/-}$)

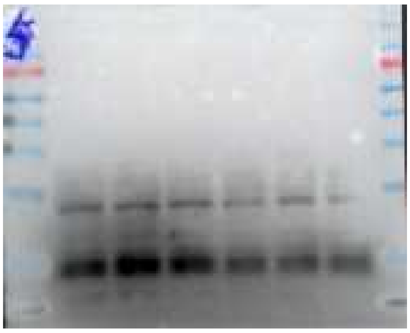

β-actin (Control vs. PC-Drp1$^{-/-}$)

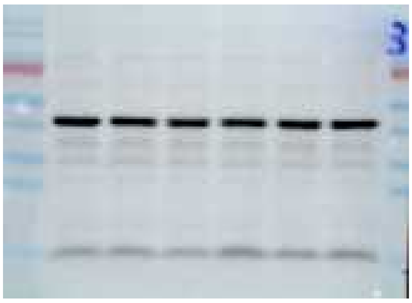

COX4 (PC-Drp1$^{-/-}$ + Veh vs. PC-Drp1$^{-/-}$ + CoQ10)

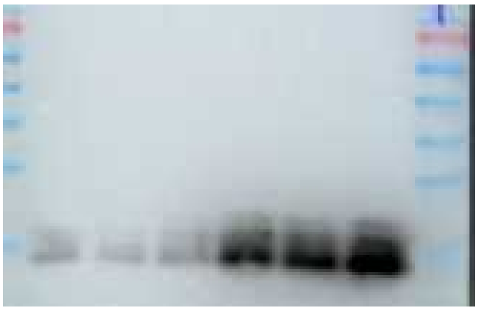

β-actin (PC-Drp1$^{-/-}$ + Veh vs. PC-Drp1$^{-/-}$ + CoQ10)

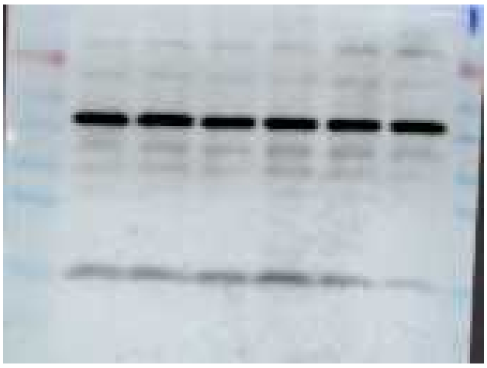

Figure 8i

SOD1 (PC-Drp1$^{-/-}$ + Veh vs. PC-Drp1$^{-/-}$ + CoQ10)

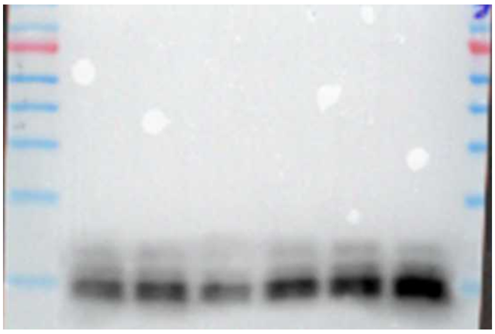

β-actin (PC-Drp1$^{-/-}$ + Veh vs. PC-Drp1$^{-/-}$ + CoQ10)

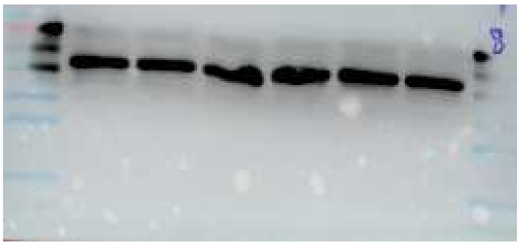

Figure 8j

GPx1 (PC-Drp1$^{-/-}$ + Veh vs. PC-Drp1$^{-/-}$ + CoQ10)

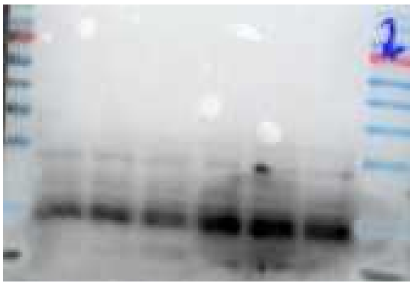

β-actin (PC-Drp1$^{-/-}$ + Veh vs. PC-Drp1$^{-/-}$ + CoQ10)

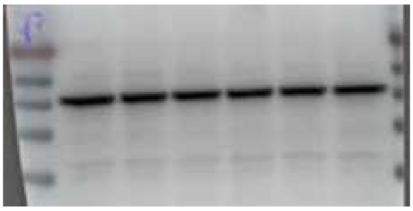

Figure 9i

CI-V (sh-Coa6-NC vs. sh-Coa6)

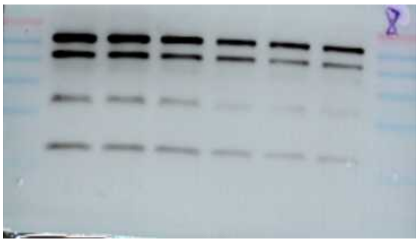

β-actin (sh-Coa6-NC vs. sh-Coa6)

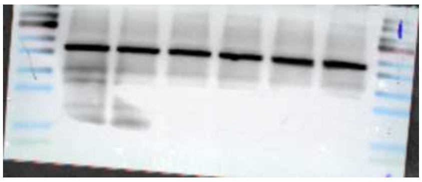

Figure 9j

COX4 (sh-Coa6-NC vs. sh-Coa6)

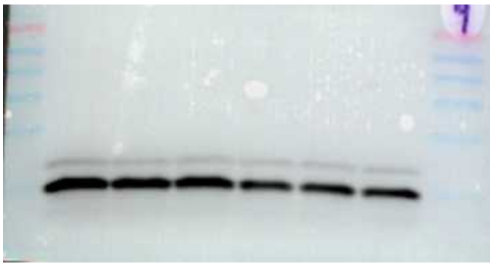

β-actin (sh-Coa6-NC vs. sh-Coa6)

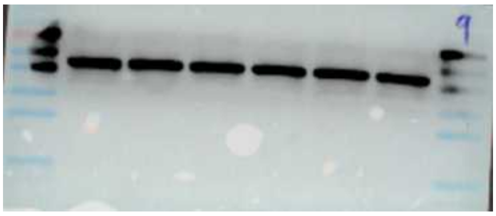

Figure 9k

GPx1 (sh-Coa6-NC vs. sh-Coa6)

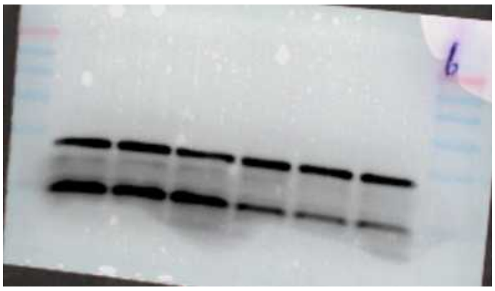

β-actin (sh-Coa6-NC vs. sh-Coa6)

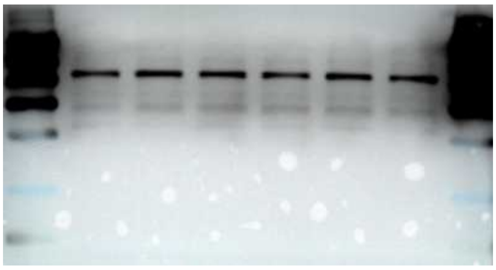

Figure 9l

SOD1 (sh-Coa6-NC vs. sh-Coa6)

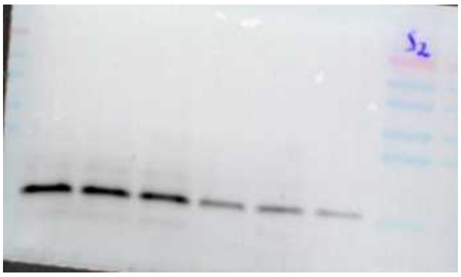

β-actin (sh-Coa6-NC vs. sh-Coa6)

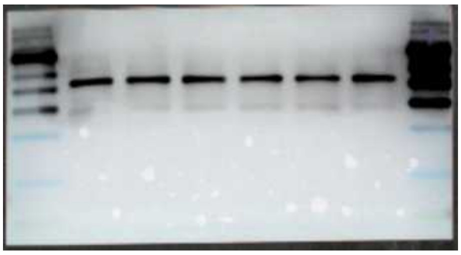

Figure 10i

CI-V (OE-Coa6-NC vs. OE-Coa6)

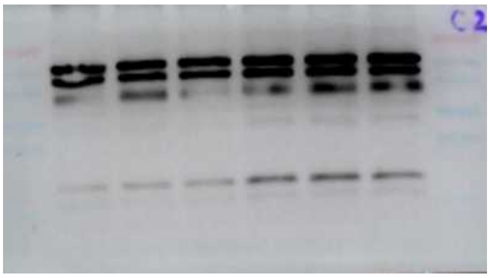

β-actin (OE-Coa6-NC vs. OE-Coa6)

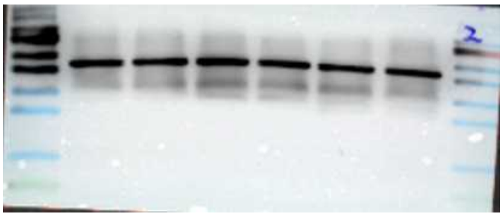

Figure 10j

COX4 (OE-Coa6-NC vs. OE-Coa6)

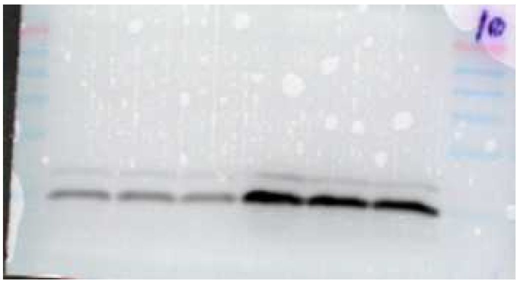

β-actin (OE-Coa6-NC vs. OE-Coa6)

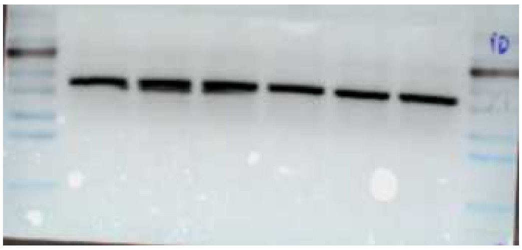

Figure 10k

GPx1 (OE-Coa6-NC vs. OE-Coa6)

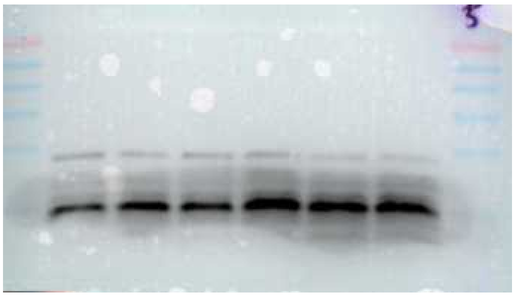

β-actin (OE-Coa6-NC vs. OE-Coa6)

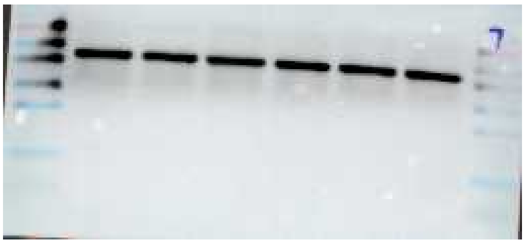

Figure 10l

SOD1 (OE-Coa6-NC vs. OE-Coa6)

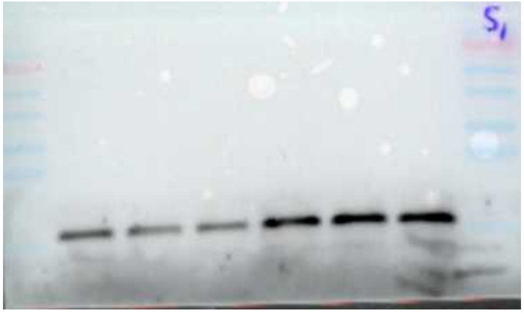

β-actin (OE-Coa6-NC vs. OE-Coa6)

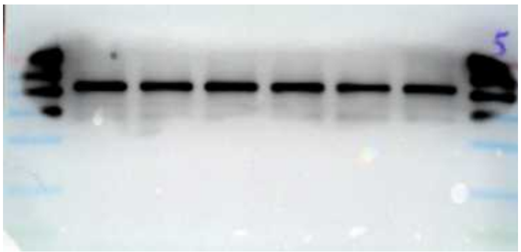

Figure S8c

Coa6 (PC-Drp1$^{-/-}$ + Veh)

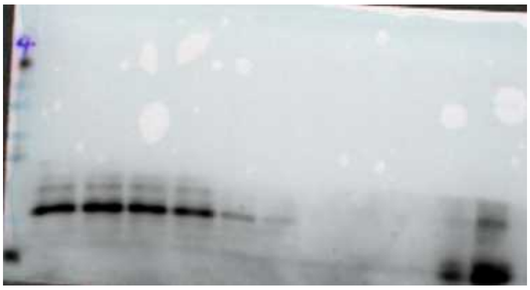

Coa6 (PC-Drp1$^{-/-}$ + CoQ10)

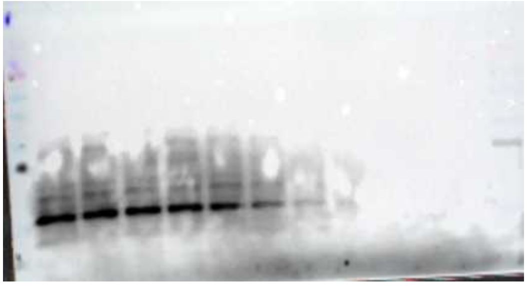

Figure S11d

Coa6 (Control)

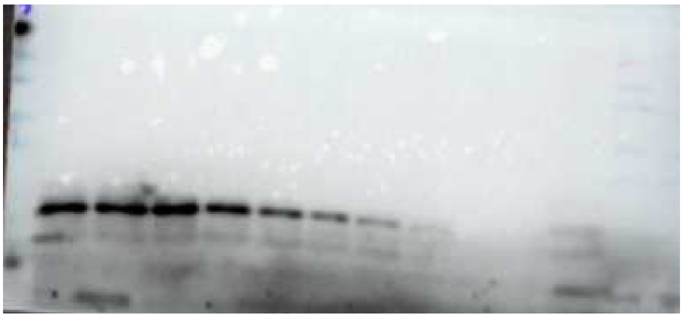

Coa6 (PC-Drp1$^{-/-}$)

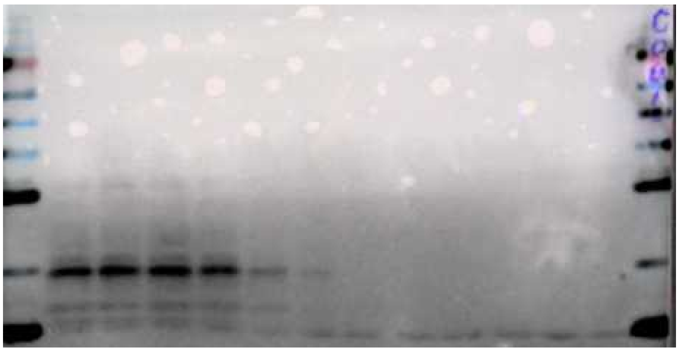

Supplement: Supplementary file 2 — Additional file 2. Full uncropped blots. [file 40035_2026_552_MOESM2_ESM.pdf]
